# Supplementary material for: Impact and cost-effectiveness of potential interventions against infant respiratory syncytial virus (RSV) in 131 low-income and middle-income countries using a static cohort model
Source: BMJ Open. 2021 Apr 24;11(4):e046563. doi: 10.1136/bmjopen-2020-046563 (PMC8074564; doi:10.1136/bmjopen-2020-046563)
Supplement: Supplementary data [file bmjopen-2020-046563supp001.pdf]

Appendix Table 1. Cost of intervention and incremental cost-effectiveness ratios by country group (2030–2039).

Country group by	Maternal vaccine			Monoclonal antibody		
	Health care cost averted	Cost of vaccination program	ICER per DALY averted	Health care cost averted	Cost of vaccination program	ICER per DALY averted
Gavi status						
Gavi (N=73)	397,985,940	3,065,106,927	1,073	1,311,275,193	3,028,242,872	315
Non-Gavi (N=58)	204,118,914	2,398,496,379	1,681	663,227,127	2,355,785,836	577
World Bank Country income group						
LIC (N=34)	131,178,798	886,636,675	949	420,421,498	867,885,189	257
LMIC (N=46)	299,612,949	2,557,430,903	1,311	995,875,548	2,522,516,388	428
UMIC (N=51)	171,313,107	2,019,535,727	1,631	558,205,274	1,993,627,132	551
Geographic region						
East Asia & Pacific (N=20)	124,384,911	1,411,293,535	1,411	392,896,831	1,380,338,857	479
Europe & Central Asia (N=20)	32,220,016	301,556,780	1,425	109,878,013	295,554,675	437
Latin America & Caribbean (N=23)	46,562,615	515,567,727	1,507	146,732,622	492,924,565	507
Middle East & North Africa (N=13)	45,216,714	496,642,134	1,566	154,303,356	504,915,613	532
South Asia (N=8)	155,092,982	1,187,286,456	1,138	558,355,660	1,236,166,379	342
Sub-Saharan Africa (N=47)	198,627,617	1,551,256,674	1,169	612,335,839	1,474,128,619	359
Total (N=131)	602,104,854	5,463,603,306	1,342	1,974,502,320	5,384,028,709	431

Abbreviations: DALY, disability-adjusted life year; ICER, incremental cost-effectiveness ratio; LIC, low-income country; LMIC, low- and middle-income country; UMIC, upper-middle-income country.

Appendix Table 2. Incremental cost-effectiveness ratio per disability-adjusted life year (DALY) averted as a percentage of gross domestic product (GDP) per capita across various scenarios.

Scenarios		Maternal vaccine						Monoclonal antibody					
		Baseline	Baseline low price	Baseline high price	Minimum	Maximum	Adjunct	Baseline	Baseline low price	Baseline high price	Minimum	Maximum	Adjunct
Efficacy (%)		Trial (40%–60%)	Trial (40%–60%)	Trial (40%–60%)	30	90	Trial (25%–39%)	60%–70%	60%–70%	60%–70%	30	90	Trial (25%–39%)
Duration of Protection (months)		3	3	3	4	6	6	6	6	6	4	6	6
Intervention cost (Gavi/non-Gavi)		$3/$5	$1.5/$2.5	$6/$10	$3/$5	$3/$5	$3/$5	$3/$5	$1.5/$2.5	$6/$10	$3/$5	$3/$5	$3/$5
Afghanistan	1	163%	82%	325%	240%	16%	7%	41%	5%	113%	234%	15%	7%
Albania		37%	21%	69%	55%	6%	17%	11%	4%	26%	55%	6%	17%
Algeria		43%	25%	79%	64%	10%	35%	15%	7%	31%	63%	9%	35%
Angola	1	36%	23%	63%	54%	7%	4%	12%	6%	24%	53%	7%	4%
Armenia	1	31%	20%	55%	47%	6%	14%	10%	5%	21%	47%	6%	14%
Azerbaijan	1	30%	19%	52%	44%	5%	2%	9%	4%	19%	44%	5%	2%
Bangladesh	1	83%	52%	145%	123%	15%	8%	27%	13%	55%	122%	15%	8%
Belarus		32%	18%	59%	46%	4%	105%	9%	3%	21%	46%	4%	104%
Belize		34%	20%	63%	51%	7%	12%	12%	5%	25%	50%	7%	12%
Benin	1	122%	63%	240%	181%	20%	11%	37%	11%	90%	177%	19%	11%
Bhutan	1	40%	25%	70%	59%	7%	8%	12%	6%	26%	58%	7%	8%
Bolivia	1	36%	23%	63%	54%	6%	7%	11%	5%	23%	53%	6%	7%
Bosnia & Herzegovina		33%	19%	61%	48%	5%	156%	10%	3%	23%	48%	5%	156%
Botswana		26%	15%	47%	39%	7%	10%	10%	5%	19%	38%	7%	10%

Brazil		19%	11%	35%	28%	4%	11%	7%	3%	14%	28%	4%	11%
Bulgaria		22%	13%	40%	32%	4%	19%	7%	3%	15%	32%	4%	18%
Burkina Faso	1	158%	80%	315%	233%	17%	10%	43%	7%	114%	232%	17%	10%
Burundi	1	333%	168%	663%	490%	33%	27%	86%	12%	235%	484%	31%	27%
Cambodia	1	90%	56%	157%	133%	14%	7%	27%	12%	58%	132%	14%	7%
Cameroon	1	93%	59%	162%	139%	19%	11%	31%	16%	61%	134%	18%	11%
Cape Verde		56%	33%	102%	83%	13%	25%	20%	9%	40%	81%	12%	24%
C.A. Rep.	1	260%	133%	513%	386%	40%	9%	76%	20%	188%	373%	37%	9%
Chad	1	148%	76%	291%	220%	25%	7%	44%	13%	107%	211%	22%	7%
China		21%	12%	38%	31%	5%	13%	8%	4%	15%	31%	5%	13%
Colombia		28%	16%	51%	41%	6%	16%	10%	5%	21%	41%	6%	16%
Comoros	1	75%	38%	148%	111%	10%	9%	20%	4%	52%	105%	9%	9%
Congo	1	71%	45%	123%	106%	15%	14%	25%	13%	49%	106%	15%	14%
Costa Rica		14%	8%	26%	21%	3%	27%	5%	2%	10%	21%	3%	27%
Cuba	1	13%	8%	23%	18%	0%	22%	2%	0%	7%	19%	0%	22%
Côte d'Ivoire	1	86%	55%	150%	128%	16%	7%	28%	14%	57%	126%	16%	7%
N. Korea	1												
D. Rep. Congo	1	212%	109%	419%	314%	30%	14%	59%	14%	150%	301%	27%	13%
Djibouti	1	61%	38%	108%	90%	7%	11%	16%	5%	37%	88%	6%	11%
Dominican Republic		25%	14%	45%	37%	5%	8%	9%	4%	18%	36%	5%	8%
Ecuador		27%	16%	49%	40%	6%	10%	10%	5%	20%	40%	6%	10%
Egypt		50%	29%	92%	74%	11%	8%	18%	8%	37%	74%	11%	8%
El Salvador		44%	26%	81%	66%	9%	22%	15%	7%	32%	64%	9%	21%
Equatorial Guinea		20%	12%	37%	30%	5%	4%	7%	4%	15%	30%	5%	4%
Eritrea	1												
Ethiopia	1	127%	65%	251%	188%	18%	15%	37%	9%	93%	185%	17%	15%
Fiji		34%	20%	63%	51%	8%	9%	12%	6%	25%	50%	8%	9%
Gabon		26%	15%	47%	38%	5%	5%	9%	4%	18%	37%	5%	5%
Gambia	1	130%	65%	260%	191%	10%	19%	32%	3%	91%	190%	10%	18%
Georgia	1	30%	19%	53%	45%	5%	42%	9%	4%	20%	44%	5%	41%

Ghana	1	63%	40%	110%	94%	12%	11%	21%	10%	42%	92%	12%	11%
Grenada		17%	10%	31%	25%	4%	8%	6%	3%	12%	25%	4%	8%
Guatemala		40%	24%	74%	60%	9%	5%	15%	7%	30%	60%	9%	5%
Guinea	1	134%	68%	265%	199%	20%	8%	40%	10%	99%	197%	19%	8%
Guinea-Bissau	1	147%	75%	291%	218%	21%	20%	43%	10%	108%	215%	20%	19%
Guyana	1	25%	16%	44%	37%	3%	10%	7%	3%	15%	36%	3%	9%
Haiti	1	123%	62%	246%	181%	10%	8%	28%	1%	83%	174%	8%	8%
Honduras	1	47%	29%	82%	69%	7%	44%	14%	6%	30%	68%	7%	43%
India	1	65%	41%	114%	96%	9%	6%	19%	7%	41%	95%	8%	6%
Indonesia	1	32%	20%	57%	48%	5%	8%	10%	4%	21%	48%	5%	8%
Iran		32%	19%	59%	47%	7%	29%	11%	5%	23%	47%	7%	29%
Iraq		38%	22%	70%	56%	8%	18%	13%	6%	27%	55%	8%	18%
Jamaica		34%	20%	63%	51%	7%	36%	12%	5%	25%	50%	7%	35%
Jordan		41%	24%	74%	60%	8%	20%	13%	6%	29%	59%	8%	19%
Kazakhstan		22%	13%	41%	32%	4%	10%	7%	2%	15%	32%	3%	10%
Kenya	1	81%	51%	141%	120%	13%	8%	25%	11%	52%	118%	12%	8%
Kiribati	1	76%	48%	132%	113%	13%	23%	24%	12%	50%	112%	13%	23%
Kyrgyzstan	1	106%	67%	184%	157%	20%	34%	35%	17%	70%	156%	20%	33%
Lao PDR	1	51%	32%	88%	76%	10%	3%	16%	8%	33%	73%	9%	2%
Lebanon		19%	11%	34%	27%	4%	54%	6%	3%	13%	27%	4%	53%
Lesotho	1	121%	76%	211%	179%	19%	10%	36%	16%	76%	173%	18%	10%
Liberia	1	130%	66%	258%	192%	15%	17%	35%	7%	93%	189%	15%	16%
Libya		40%	24%	74%	60%	8%	109%	14%	6%	29%	59%	8%	108%
Madagascar	1	234%	120%	462%	346%	33%	14%	66%	16%	167%	335%	30%	14%
Malawi	1	307%	154%	614%	449%	21%	36%	70%	1%	207%	438%	18%	35%
Malaysia		17%	10%	32%	26%	4%	27%	6%	3%	13%	26%	4%	27%
Maldives		16%	9%	29%	23%	4%	52%	6%	3%	12%	23%	4%	52%
Mali	1	125%	65%	247%	186%	21%	12%	39%	11%	93%	183%	20%	12%
Mauritania	1	117%	74%	203%	174%	24%	15%	38%	19%	76%	167%	22%	15%
Mauritius		17%	10%	31%	25%	4%	18%	6%	3%	13%	25%	4%	18%
Mexico		20%	11%	36%	29%	4%	11%	7%	3%	14%	29%	4%	11%
Micronesia		55%	32%	100%	81%	13%	23%	20%	10%	40%	81%	13%	23%
Mongolia	1	33%	21%	57%	48%	6%	4%	11%	5%	21%	48%	6%	4%

Montenegro		23%	13%	42%	33%	3%	108%	7%	2%	15%	33%	3%	108%
Morocco		58%	34%	106%	85%	12%	24%	20%	9%	41%	84%	12%	24%
Mozambique	1	245%	124%	487%	361%	28%	34%	67%	12%	177%	360%	27%	34%
Myanmar	1	98%	62%	169%	144%	18%	9%	32%	16%	64%	144%	18%	9%
Namibia		39%	23%	72%	58%	9%	7%	14%	6%	29%	58%	9%	7%
Nepal	1	109%	54%	221%	160%	3%	11%	23%	-2%	73%	159%	3%	11%
Nicaragua	1	52%	33%	91%	77%	8%	15%	16%	7%	33%	76%	8%	15%
Niger	1	266%	135%	529%	392%	29%	20%	68%	10%	185%	379%	26%	19%
Nigeria	1	63%	40%	109%	93%	14%	3%	21%	11%	42%	91%	13%	3%
Pakistan	1	86%	54%	151%	127%	13%	9%	24%	10%	52%	120%	11%	8%
Papua New Guinea	1	51%	33%	89%	76%	10%	8%	17%	8%	34%	75%	10%	8%
Paraguay		31%	18%	57%	46%	6%	23%	10%	5%	22%	45%	6%	23%
Peru		27%	16%	50%	40%	6%	9%	10%	5%	20%	40%	6%	9%
Philippines		58%	34%	106%	85%	12%	10%	20%	9%	42%	85%	12%	10%
Republic of Moldova	1	58%	37%	102%	86%	9%	12%	18%	8%	38%	86%	9%	12%
Romania		17%	10%	31%	25%	3%	7%	5%	2%	12%	25%	3%	7%
Russian Federation		19%	11%	35%	28%	3%	25%	6%	2%	13%	28%	3%	25%
Rwanda	1	127%	65%	252%	188%	15%	16%	34%	6%	90%	183%	14%	15%
Saint Lucia		18%	10%	33%	26%	4%	13%	6%	3%	13%	26%	4%	13%
St. Vincent & Grenad.		23%	14%	43%	35%	5%	11%	8%	4%	17%	35%	5%	11%
Samoa		45%	26%	81%	66%	11%	26%	16%	8%	33%	65%	11%	26%
S. Tome & Principe	1	68%	43%	118%	100%	11%	13%	20%	9%	43%	98%	10%	13%
Senegal	1	73%	37%	146%	108%	7%	10%	19%	3%	52%	106%	7%	10%
Serbia		29%	17%	55%	43%	4%	114%	8%	3%	20%	43%	4%	113%
Sierra Leone	1	194%	97%	388%	285%	15%	11%	47%	3%	134%	282%	14%	10%
Solomon Islands	1	60%	38%	105%	89%	11%	15%	19%	9%	38%	86%	10%	15%
Somalia	1	217%	113%	425%	323%	41%	17%	69%	23%	161%	310%	38%	16%

South Africa		33%	19%	61%	49%	7%	8%	11%	5%	24%	48%	7%	7%
South Sudan	1	396%	196%	797%	580%	20%	14%	85%	-4%	264%	564%	17%	14%
Sri Lanka	1	30%	19%	52%	44%	6%	67%	10%	5%	20%	44%	6%	66%
State of Palestine		59%	35%	108%	89%	16%	124%	23%	12%	45%	88%	16%	123%
Sudan	1	51%	32%	89%	75%	9%	9%	16%	8%	33%	74%	9%	9%
Suriname		30%	18%	55%	44%	7%	10%	11%	5%	22%	44%	6%	10%
Swaziland		64%	38%	117%	95%	14%	7%	23%	11%	47%	95%	14%	7%
Syria													
Macedonia		31%	18%	57%	45%	4%	45%	9%	3%	21%	45%	4%	44%
Tajikistan	1	116%	60%	230%	172%	16%	7%	34%	8%	85%	171%	16%	7%
Thailand		28%	16%	51%	41%	6%	32%	10%	5%	20%	41%	6%	32%
Timor-Leste	1	60%	38%	104%	89%	11%	7%	19%	9%	38%	86%	10%	7%
Togo	1	162%	82%	321%	239%	19%	22%	44%	8%	115%	234%	18%	21%
Tonga		47%	28%	86%	70%	11%	24%	17%	8%	35%	69%	11%	23%
Tunisia		46%	27%	84%	68%	10%	83%	16%	8%	33%	67%	10%	83%
Turkey		15%	9%	27%	22%	3%	25%	5%	2%	11%	22%	3%	25%
Turkmenistan		27%	16%	50%	40%	5%	4%	9%	4%	19%	40%	5%	4%
Uganda	1	157%	78%	315%	230%	9%	29%	35%	0%	105%	224%	8%	29%
Ukraine	1	52%	33%	90%	76%	8%	89%	16%	7%	33%	76%	8%	89%
Tanzania	1	103%	52%	203%	151%	12%	10%	27%	5%	72%	146%	11%	10%
Uzbekistan	1	56%	35%	97%	83%	10%	5%	18%	9%	37%	82%	10%	5%
Vanuatu		62%	37%	113%	92%	15%	10%	23%	11%	46%	91%	15%	10%
Venezuela													
Viet Nam	1	51%	32%	89%	75%	9%	21%	16%	8%	34%	75%	9%	21%
Yemen	1	69%	35%	137%	102%	10%	13%	20%	5%	50%	100%	9%	13%
Zambia	1	99%	62%	173%	146%	15%	11%	29%	13%	62%	143%	14%	11%
Zimbabwe	1	74%	37%	146%	109%	9%	5%	21%	4%	54%	108%	9%	5%

Note: Country by Gavi status (Gavi country = 1). Current GDP values were not available for 4 countries (Venezuela, Syria, Eretria, North Korea) and were excluded from the analysis.

*Negative cost-effectiveness ratio implies cost savings.

Cells in green indicate lower ICER to GDP ratio and cells in red indicate higher ICER to GDP ratio.
