# Supplementary material for: Impact and cost-effectiveness of potential interventions against infant respiratory syncytial virus (RSV) in 131 low-income and middle-income countries using a static cohort model
Source: BMJ Open. 2021 Apr 24;11(4):e046563. doi: 10.1136/bmjopen-2020-046563 (PMC8074564; doi:10.1136/bmjopen-2020-046563)
Supplement: Author's manuscript [file bmjopen-2020-046563.draft_revisions.pdf]

BMJ Open is committed to open peer review. As part of this commitment we make the peer review history of every article we publish publicly available.

When an article is published we post the peer reviewers' comments and the authors' responses online. We also post the versions of the paper that were used during peer review. These are the versions that the peer review comments apply to.

The versions of the paper that follow are the versions that were submitted during the peer review process. They are not the versions of record or the final published versions. They should not be cited or distributed as the published version of this manuscript.

BMJ Open is an open access journal and the full, final, typeset and author-corrected version of record of the manuscript is available on our site with no access controls, subscription charges or pay-per-view fees (http://bmjopen.bmj.com).

If you have any questions on BMJ Open's open peer review process please email info.bmjopen@bmj.com

BMJ Open

Impact and cost-effectiveness of potential interventions against infant respiratory syncytial virus (RSV) in low- and middle-income countries

Journal:	*BMJ Open*
Manuscript ID	bmjopen-2020-046563
Article Type:	Original research
Date Submitted by the Author:	03-Nov-2020
Complete List of Authors:	Baral, Ranju; Program for Appropriate Technology in Health, Center for Vaccine Innovation and Access<br>Higgins , Deborah ; Program for Appropriate Technology in Health, Center for Vaccine Innovation and Access<br>Regan, Katie; Program for Appropriate Technology in Health, Center for Vaccine Innovation and Access<br>Pecenka, Clint; Program for Appropriate Technology in Health, Center for Vaccine Innovation and Access
Keywords:	Health economics < HEALTH SERVICES ADMINISTRATION & MANAGEMENT, PAEDIATRICS, Respiratory infections < THORACIC MEDICINE

SCHOLARONE™
Manuscripts

I, the Submitting Author has the right to grant and does grant on behalf of all authors of the Work (as defined in the below author licence), an exclusive licence and/or a non-exclusive licence for contributions from authors who are: i) UK Crown employees; ii) where BMJ has agreed a CC-BY licence shall apply, and/or iii) in accordance with the terms applicable for US Federal Government officers or employees acting as part of their official duties; on a worldwide, perpetual, irrevocable, royalty-free basis to BMJ Publishing Group Ltd ("BMJ") its licensees and where the relevant Journal is co-owned by BMJ to the co-owners of the Journal, to publish the Work in this journal and any other BMJ products and to exploit all rights, as set out in our licence.

The Submitting Author accepts and understands that any supply made under these terms is made by BMJ to the Submitting Author unless you are acting as an employee on behalf of your employer or a postgraduate student of an affiliated institution which is paying any applicable article publishing charge ("APC") for Open Access articles. Where the Submitting Author wishes to make the Work available on an Open Access basis (and intends to pay the relevant APC), the terms of reuse of such Open Access shall be governed by a Creative Commons licence – details of these licences and which Creative Commons licence will apply to this Work are set out in our licence referred to above.

Other than as permitted in any relevant BMJ Author's Self Archiving Policies, I confirm this Work has not been accepted for publication elsewhere, is not being considered for publication elsewhere and does not duplicate material already published. I confirm all authors consent to publication of this Work and authorise the granting of this licence.

For peer review only

Impact and cost-effectiveness of potential interventions against infant respiratory syncytial virus (RSV) in low- and middle-income countries

Authors:

Ranju Baral, Center for Vaccine Innovation and Access, PATH, Seattle, USA

Deborah Higgins, Center for Vaccine Innovation and Access, PATH, Seattle, USA

Katie Regan, Center for Vaccine Innovation and Access, PATH, Seattle, USA

Clint Pecenka, Center for Vaccine Innovation and Access, PATH, Seattle, USA

Corresponding author:

Ranju Baral, Health Economist

Center for Vaccine Innovation and Access, PATH, Seattle, USA

rbaral@path.org

206-285-3500

Running title:

Infant RSV interventions in LMIC

For peer review only

For peer review only - http://bmjopen.bmj.com/site/about/guidelines.xhtml

Abstract

Background. Interventions to prevent childhood respiratory syncytial virus (RSV) disease are limited and costly. New interventions are in advanced stages of development and could be available for soon. We evaluate the potential impact and cost-effectiveness of two such interventions—a maternal vaccine and a monoclonal antibody (mAb).

Methods. Using a static population-based cohort model, we evaluate impact and cost-effectiveness of RSV interventions across 131 low- and middle-income countries, from a health systems perspective. The assumed baseline efficacy and duration of protection were higher for the mAb compared to the maternal vaccine. Both interventions were evaluated at US$3 and $5 per dose for Gavi and non-Gavi countries, respectively. A range of input values were considered to explore uncertainty.

Results. Under baseline assumptions, maternal vaccine and mAbs were projected to avert 25% and 55% of RSV-related deaths among infants younger than six months of age, respectively. The average incremental cost-effectiveness ratio per disability-adjusted life year averted was $1,342 (range $800 to $1,866) for maternal RSV vaccine and $431 (range $167 to $692) for mAbs. At a 50% gross domestic product per capita threshold, maternal vaccine and mAbs were cost-effective in 60 and 118 countries, respectively.

Conclusions. Both interventions are projected to be impactful and cost-effective in many countries, a finding that would be enhanced if country-specific Gavi co-financing to eligible countries were included. mAbs, with assumed higher efficacy and duration of protection, are expected to be more cost-effective than RSV maternal vaccines at similar prices. Final product characteristics will influence this finding.

Key words: Respiratory syncytial virus (RSV); maternal RSV vaccine; RSV monoclonal antibody; health impact; cost-effectiveness.

For peer review only - http://bmjopen.bmj.com/site/about/guidelines.xhtml

Strengths and limitations of this study

- This is one of the first studies to examine the potential impact and cost-effectiveness of maternal vaccines and monoclonal antibodies for RSV prevention, across 131 low-and middle-income countries.
- This study compares products with uncertain characteristics using the latest available data on vaccine characteristics, supplemented by the target product profile to inform the model parameters.
- A range of input values were considered to explore uncertainty, insights from which are useful to inform subsequent intervention development.
- Final product characteristics and product prices will determine the relative cost effectiveness of RSV interventions.

For peer review only

For peer review only - http://bmjopen.bmj.com/site/about/guidelines.xhtml

Introduction

Respiratory syncytial virus (RSV) is a common cause of acute lower respiratory illness (ALRI) among children younger than age five, causing between 41,000 and 118,000 child deaths per year globally [1,2]. RSV disease is most severe among young infants, and the burden is highest in low- and middle-income countries (LMICs), where more than 99% of RSV deaths occur [2]. Emerging evidence indicates the unrecognized burden of RSV among children in low-resource settings is also significant, with up to 10% of young infant deaths attributable to RSV infection [3,4].

Existing RSV interventions are limited and cost prohibitive, even in high-income countries [5]. Several prophylactic interventions are currently under development [6,7]. Multiple maternal vaccine candidates designed to protect against RSV illness in young children are in relatively advanced stages of development and expected to be available for global use in the coming years [6]. Monoclonal antibodies (mAbs) are available and in use for high-risk babies in high-income countries, but more affordable mAbs are also in advanced stages of development [8]. Given the extent of the global RSV disease burden—especially in low-income countries (LICs)—and the lack of efficacious and cost-effective therapeutic options, these new interventions are expected to be included in Gavi, the Vaccine Alliance's, portfolio [9], subject to licensure, prequalification, and cost characteristics.

In this paper, we estimate the potential impact and cost-effectiveness of a maternal vaccine and a mAb, both designed to avert RSV disease burden in young infants in LMICs. We compare each intervention against a scenario of no intervention and against each other. Results from this study illustrate the potential benefits of these products and will help inform decisions around further development. This analysis will also inform global and LMIC decision-makers likely to face choices about whether and which interventions to introduce.

Methods

We examined the potential impact and cost-effectiveness of a single-dose RSV maternal vaccine administered to pregnant women at 24 to 36 weeks gestation, and of a single-dose mAb given to infants

For peer review only - http://bmjopen.bmj.com/site/about/guidelines.xhtml

directly at birth across 131 LMICs, compared to no intervention. Both interventions were evaluated independently using a static cohort model. For maternal vaccine, infants born two weeks following maternal immunization were considered as protected to allow time for immune transfer from mother to children. All children receiving mAbs were considered protected immediately.

We examined the impact and cost of interventions from the health systems perspective over the period 2030 to 2039 (10 years), assuming nationwide introduction in 2030. Primary input values for a baseline scenario are given in Table 1. Key model outputs include cases averted, severe cases averted, hospitalizations averted, deaths averted, disability adjusted life years (DALYs) averted, and the incremental cost per DALY averted due to RSV interventions. Given the inconsistent use of country-specific cost-effectiveness thresholds across LMICs, we used a willingness-to-pay threshold of 0.5 times the gross domestic product (GDP) per capita in each country [10]. Results are summarized by World Health Organization (WHO) regions, World Bank income group, and Gavi eligibility to understand impact by country group. All monetary units are adjusted to 2016 US dollars.

[Insert Table 1 here: Key parameter values used for modeling]

Disease burden

Disease burden inputs including disease incidence, severe disease incidence, incidence of hospitalizations, and mortality were derived from a comprehensive systematic review paper [2]. We combined country-specific disease incidence estimates in children under five years of age with a representative developing-country estimate to generate incidence by granular age band in each country. To generate incidence of severe disease, hospitalization, and hospital mortality, we used developing-country estimates [2]. Estimated hospital deaths were adjusted by multiplying by 1.98 to account for community deaths and influenza coinfection [2]. The actual values of disease burden inputs are given in Table 1.

Some RSV interventions under development have shown promising results in their ability to avert all-cause lower respiratory tract infections (LRTIs) among children [11], in addition to RSV infection. Thus, we also explored the potential impact of both RSV interventions on all-cause LRTI, based on emerging burden data, using estimates from the Global Burden of Disease Study 2017 [12], and assuming a uniform

For peer review only

For peer review only - http://bmjopen.bmj.com/site/about/guidelines.xhtml

distribution of disease among children one to 12 months of age. Further, we assumed 11.5% of all-cause LRTI cases would result in severe cases [13] and 40% of all severe cases would result in hospitalization.

Intervention introduction and coverage

The leading RSV intervention candidates could be available for use in the next five-to-eight years [7]. We assumed both interventions would be available by 2030 and all countries would begin national introduction in 2030.

All pregnant women attending antenatal care (ANC) visits were assumed eligible to receive RSV maternal vaccine. To project the number of pregnant women per country, we added country-specific stillbirths to the United Nations Population Division annual birth projections [14]. We estimated maternal vaccine coverage during the 24- to 36-week vaccination window by examining country-specific ANC first-visit timing [15], country-specific ANC coverage [16], and the WHO's recommended ANC timing based on the focused ANC model guideline [17]. Details on methods used in estimating maternal vaccine coverage during ANC within a specific gestation window is described elsewhere [18].

All live births were considered eligible for mAbs. All infants were assumed to be covered at the Bacillus Calmette–Guérin (BCG) vaccine birth dose coverage levels adjusted to the timeliness of vaccine receipt. Country's overall BCG coverage were derived from the most recent WHO/UNICEF estimates of national immunization coverage [19]. Timeliness of BCG birth dose receipt was derived using the methods described in the literature [20,21].

Coverage levels for both interventions for each country are projected to improve by 3 percentage points each year until coverage reaches 70%, and after that by 1 percentage point each year until reaching 95% coverage. This projection was made to correspond with methods applied during the Gavi vaccine investment strategy [9].

Intervention characteristics

Our analysis assumed a single dose maternal RSV vaccine would be given to pregnant women between 24 and 36 months of gestation, based on the WHO preferred product characteristics (PPCs) [22]. We based vaccine efficacy and duration of protection on data from one of the first maternal vaccine candidate

For peer review only - http://bmjopen.bmj.com/site/about/guidelines.xhtml

Phase 3 clinical trials (Table 1) [11]. Given the uncertainty in vaccine characteristics, scenario analyses included a range in efficacy (30% to 90%) and duration of protection afforded to infants (three to six months).

Our analysis assumed a single dose mAb would be given to newborns at birth, would have 60% to 70% efficacy, and would offer protection for six months, based on the PPCs [23]. As with the maternal vaccine, we varied efficacy and duration of protection in scenario analysis. We assumed neither intervention contributed to herd immunity, and that efficacy did not wane during the period of protection.

Intervention price and delivery costs

For both interventions, we assumed a per-dose price of US$3 in Gavi-eligible countries and per-dose price of $5 in LMICs not eligible for Gavi support. If Gavi decides to support RSV interventions once they are available, Gavi-eligible countries would likely be able to access the interventions at varying prices depending on their transition status [24]. We refrained from projecting individual country Gavi eligibility or intervention prices due to significant uncertainty, and instead evaluated a range of intervention prices in sensitivity analyses.

Given the paucity of data on maternal immunization and mAb delivery costs in LMICs, we used delivery costs estimates for other vaccines derived from the Immunization Costing Action Network (ICAN) repository [25]. Unit costs of delivering RSV interventions were $0.63 for LICs and $1.73 for LMICs. We accounted for vaccine/mAbs wastage at 5% and a buffer stock at 25% of demand in the introduction year, and at 25% of the incremental demand in subsequent years.

Health service costs

Very few studies have analyzed the cost of managing RSV in children, especially in LMICs [26–31]. Hospitalization costs also vary widely. In Bangladesh, for example, hospitalization averages $74, whereas in China it averages $662. Given limited RSV-specific information in LMICs, we used the average cost of treating pneumonia in young children, identified in a systematic review [32] as $53.26 and $250.04 per outpatient and inpatient episode, respectively. We assumed that severe cases seek inpatient care and non-severe cases seek outpatient care.

For peer review only

For peer review only - http://bmjopen.bmj.com/site/about/guidelines.xhtml

Cost-effectiveness analysis

We calculated intervention costs by multiplying the number of doses (estimated number of pregnant women receiving vaccine for maternal vaccine and estimated number of live births for mAbs) with the unit cost of delivery and cost per dose. We estimated averted health care costs by multiplying the estimated number of non-severe/severe cases averted by the costs of an outpatient/inpatient episode. Vaccine impact was calculated by multiplying the respective disease burden in children born two weeks after maternal vaccination with vaccine efficacy. The mAb impact was calculated by multiplying disease burden with the BCG coverage estimates and mAb efficacy. We estimated health outcomes including severe/non-severe cases averted, hospitalizations averted, deaths averted, and DALYs averted for each country and year. Disability weights for non-severe and severe ALRI were used to compute DALYs [33]. Further, we assumed duration of illness at five days for non-severe disease and 10 days for severe disease [34]. The length of a hospital stay for severe disease was assumed to be 6.4 days [32]. We also accounted for variation in health-seeking practices by using health care use data from children younger than five years receiving pneumonia care [35].

We calculated incremental cost-effectiveness ratios (ICERs) for each country by dividing the net cost of intervention by the net DALYs averted by the intervention.

Sensitivity analysis

We conducted one-way sensitivity analysis by changing the values of key input parameters, including intervention efficacy, duration of protection, anticipated coverage, and intervention price. Alternate scenarios that changed one or more input parameters to evaluate results sensitivity were also considered. In an adjunct scenario, we evaluated how different interventions show impact on all-cause LRTI mortality, using the efficacy and duration of protection values as suggested by recent clinical trial data [11], and disease burden for all-cause LRTI from the 2017 Global Burden of Disease Study [12].

Results

For peer review only - http://bmjopen.bmj.com/site/about/guidelines.xhtml

Disease burden without interventions

Over the 10-year period, about 41.94 million non-severe cases, 15.28 million severe cases, 11.48 million hospitalizations, and 504,963 thousand deaths among children younger than six months of age in 131 LMICs are projected (Table 3). Seventy-three Gavi-eligible countries accounted for 70% of the mortality burden. Most deaths would occur in sub-Saharan Africa (36%, 47 countries), followed by South Asia (26%, eight countries).

[Insert Table 2 here: Summary of disease burden, impact and cost-effectiveness ratios with and without intervention (2030-2039), baseline scenario]

Expected health outcomes with intervention

RSV maternal vaccine, under the baseline scenario, has the potential to avert 2.97 million non-severe cases, 2.63 million severe cases, 2.03 million hospitalizations, 126,552 deaths, and 3.73 million DALYs (discounted) among children younger than six months of age across all countries over 10 years (Table 2). Globally, about 17% of severe RSV cases and 25% of RSV-related deaths among infants under six months of age would be averted by RSV maternal vaccine, which is roughly 13 deaths averted per 100,000 vaccinated pregnant women.

An RSV mAb, under the baseline scenario, is expected to avert 19.47 million cases of non-severe disease, 7.18 million severe cases, 5.40 million hospitalizations, 276,933 deaths, and 8.19 million DALYs (discounted) among children younger than six months of age across all countries over 10 years (Table 2). Globally, about 55% of RSV deaths among infants younger than six months of age would be averted with RSV mAbs—equivalent to approximately 28 averted deaths per 100,000 newborns receiving the intervention.

Under alternative scenarios using varying efficacy and duration of protection assumptions (minimum and maximum scenarios), the RSV maternal vaccine is estimated to avert between 84,934 and 356,346 deaths over 10 years; and the RSV mAb is expected to avert roughly 84, 864 and 356, 057 deaths. Assuming both interventions are able to affect all-cause LRTI, as suggested by recent clinical trial data [11], either

For peer review only - http://bmjopen.bmj.com/site/about/guidelines.xhtml

intervention is projected to avert roughly 1.05 million LRTI deaths (29% of all LRTI deaths) among children younger than six months of age in LMICs.

Cost-effectiveness of interventions

The average annual cost of vaccination programs across all countries for the duration of analysis was estimated to be about $546.36 million and $538.40 million for RSV maternal vaccine and mAbs, respectively. The economic benefits expressed in terms of cost-of-care averted was about $602.10 million (maternal vaccine) and $1.97 billion (mAbs) over the 10 years (see appendix Table 1).

For maternal RSV vaccine, the ICER per DALY averted is estimated at $1,342 ($1,073 across Gavi-eligible countries and $1,681 across non-Gavi countries). Similarly, the ICER estimates for RSV mAbs is $431 ($315 across Gavi-eligible countries and $577 across non-Gavi countries). It is important to note these ICERs reflect the full potential cost of either intervention. Countries eligible for Gavi support would be expected to pay a share of the prices used in this analysis, thus reducing the ICER from the country perspective.

Results from alternative scenarios with low and high efficacy and duration of protection assumptions show that costs per DALY averted across countries range from $244 to $1,982 (maternal vaccine) and $239 to $1,958 (mAbs). By reducing the intervention price to 50% of the baseline price (i.e., $1.50 for Gavi-eligible countries and $2.50 for non-Gavi countries), the average ICER per DALY averted would decline to $781 (range $45 to $1,147) for the maternal vaccine and $178 (range $42 to $1,132) for the mAb. Increasing the intervention price by 200% of the baseline price, the average ICER per DALY averted increases to $2,465 (range $642 to $3,651) for the maternal vaccine and $938 (range $632 to $3,610) for the mAbs.

When comparing ICERs against an individual country's income level at baseline, the maternal vaccine ICERs were less than 50% of the GDP per capita in 60 countries (12 Gavi and 48 non-Gavi), suggesting intervention cost-effectiveness in those countries. ICERs for RSV mAbs were below the 50% GDP per capita threshold in 118 countries (62 Gavi-eligible and all non-Gavi). For both interventions, countries with higher ICER to GDP per capita ratios are concentrated in sub-Saharan Africa and Asia (Figure 1).

For peer review only - http://bmjopen.bmj.com/site/about/guidelines.xhtml

Many of these countries remain eligible for Gavi support and are expected to pay lower intervention prices. As a result, the cost per DALY averted from the perspective of these countries is likely to be much more favorable than shown here. For example, if each of the original Gavi-eligible countries were responsible for half of the cost of the intervention ($1.50), which is still a relatively high cost as the countries with the lowest GDP per capita would pay only a fraction of that price under Gavi's current co-financing model, then the ICER for the RSV maternal vaccine and mAb would fall below the 50% GDP per capita threshold in 46% (maternal vaccine) and 100% (mAb) of these countries. Further, maternal vaccine ICERs across countries at base price are roughly equivalent to the mAb ICER evaluated at 300% of the base price. Appendix Table 2 includes a comparison of ICERs as a share of country GDP for alternative intervention scenarios.

[Insert Figure 1 here: Incremental cost-effectiveness ratios as a percentage of national GDP per capita.]

Discussion

Both RSV interventions are projected to be impactful across all countries under baseline assumptions. A maternal vaccine is projected to avert 12.65 thousand deaths and mAbs roughly two times more (27.69 thousand deaths averted) annually among children younger than six months of age. We note that our baseline assumptions for the maternal vaccine draw from a Phase 3 trial in which the primary endpoints were not met. As a result, maternal vaccine assumptions may be conservative compared to mAb assumptions, leading to lower overall impact of RSV maternal vaccines. Under alternative scenarios that consider both interventions with similar characteristics, we observe no substantial variation in impact. Under a minimal (30% efficacy and four months protection) and maximal (90% efficacy and six months protection) intervention characteristics scenario, both interventions are projected to avert roughly 84,900 and 356,000 deaths among children younger than six months of age across 131 countries, suggesting that efficacy and duration of protection are primary parameters for determining impact, reinforced by a similar study [36].

For peer review only - http://bmjopen.bmj.com/site/about/guidelines.xhtml

Unknowns around intervention delivery strategy and potential coverage implications create uncertainties; this is especially true for a novel intervention like a maternal vaccine. To further understand the potential implications of unknown parameters on a maternal vaccine impact, we evaluated the marginal gains in impact by incrementally changing the parameter values to mimic those used in the mAb baseline scenario. When changing maternal vaccine coverage assumptions to the mAb coverage values, the maternal vaccine would prevent 22,000 additional deaths. Similarly, when changing both duration of protection and efficacy for maternal vaccine at baseline to the mAb baseline equivalent, maternal vaccine would avert an additional 150,000 deaths. As seen in Figure 2, the duration of protection is the most important factor for increasing impact (109,000 additional deaths averted).

[Insert Figure 2 here: Impact of change in key input parameter values on deaths averted.]

The cost per DALY averted under the baseline scenario for a maternal vaccine is more than three times that for mAbs ($1,342 versus $431). This is mainly driven by the modest vaccine efficacy and assumed duration of protection for the maternal vaccine as compared to mAbs. Under the maximum and minimum scenarios with high and low vaccine efficacy and duration of protection assumptions, the difference in the estimated ICERs between the two interventions is muted (Figure 3).

[Insert Figure 3 here: Average incremental cost-effectiveness ratios by country groups.]

Though it didn’t meet the primary endpoint, the recent Phase 3 maternal vaccine trial shows promising impact on all-cause LRTI mortality [11]. If both future RSV interventions reduce all-cause LRTI mortality, our adjunct scenario shows more pronounced impact by averting more than a million all-cause LRTIs during the 10-year period. ICER estimates under this scenario were $896 for the maternal vaccine (range $34 to $7,602) and $889 for the mAb (range $33 to $7,608) per DALY averted across all countries, with 116 countries (69 Gavi-eligible and 47 non-Gavi countries) demonstrating ICERs less than 50% of their respective GDP per capita. We refrained from directly comparing these estimates to

For peer review only - http://bmjopen.bmj.com/site/about/guidelines.xhtml

other scenarios as they use data sources [12] not comparable with the primary disease burden data [2] used in other scenarios.

There are several additional limitations worth citing. There is a dearth of RSV disease burden data, especially regarding the age distribution of disease in young infants in LMICs. Although we used the best published estimates of RSV disease burden in children [2], the literature is expanding rapidly. For example, studies from Zambia [37] and Argentina [38] highlight that community mortality and deaths from RSV could be as high as 10% and 11% of all-cause deaths occurring among infants up to six months of age. This highlights a large and underappreciated burden of RSV and would mean our estimates of impact and effectiveness are conservative. Although we attempted to quantify the potential benefits of RSV interventions with additional scenario analysis, lack of consistent input data coupled with poorly established age distribution limits the comparability of our results across these scenarios. Collecting more granular data on disease burden is critical to inform future studies [36].

The products evaluated in this study are not yet available in the market so other key parameters are unknown. We assumed the same price for both interventions, which may not hold, as historical evidence suggests mAbs are likely to be more expensive to produce than a vaccine [5]. This could have considerable impact on the ICERs and comparisons between products. Nonetheless, our analysis shows that the mAb is more cost-effective than a maternal vaccine at baseline efficacy and duration of protection values, until a mAb reaches approximately three times the baseline price assumption. Gavi evaluated both interventions for inclusion in its 2018 Vaccine Investment Strategy in anticipation of the potential benefits, and they are expected to be included in the Gavi portfolio, subject to licensure, prequalification, and affordability. In that case, the eligible Gavi countries would benefit from a considerable subsidy for access and affordability, especially the countries with the lowest GPDs per capita. Further, the <50% of GDP per capita thresholds used in this paper are non-specific measures of cost-effectiveness, especially when intervention prices to be paid by individual Gavi-supported countries are not yet known. Country-specific thresholds are recommended [39] but often do not exist for most LMICs. In the absence of

For peer review only - http://bmjopen.bmj.com/site/about/guidelines.xhtml

country-specific thresholds, we used a conservative metric uniform across all countries to define cost-effectiveness.

Lastly, RSV infection is seasonal in many countries. We did not consider seasonal delivery in this analysis. Seasonal intervention could potentially be a more cost-effective yet feasible strategy [40], especially when using mAbs to selectively immunize children before the start of the RSV season. Delivering maternal vaccine seasonally to pregnant woman in LMICs may be more challenging due to the lack of a defined maternal vaccine delivery strategy. Future research should explore the feasibility of alternative delivery strategies.

Conclusions

RSV interventions evaluated in this study are projected to be impactful and cost-effective across many LMICs. Under the assumptions used, mAbs are comparatively more impactful and cost-effective than RSV maternal vaccines. However, we reiterate the uncertainty around several critical parameters that inform this finding. The emerging evidence of RSV's role in all LRTI deaths among young infants suggests our analyses of RSV burden averted may prove conservative and enhance the attractiveness of RSV interventions as important tools for curbing LRTI mortality in infants. As disease burden shifts toward neonates and very young children, RSV maternal immunization and mAbs offer the opportunity to protect young infants from disease.

Funding

This work was funded by a grant from the Bill & Melinda Gates Foundation [OPP1088264], Seattle, WA, USA. The findings and conclusions contained within are those of the authors and do not necessarily reflect positions or policies of the Bill & Melinda Gates Foundation.

Acknowledgments

For peer review only - http://bmjopen.bmj.com/site/about/guidelines.xhtml

We would like to thank Susan Nazarro for her critical feedback on this manuscript.

Author contributions

CP, DH and RB conceptualized the study. RB and CP developed the model. RB performed the analysis. RB and CP wrote the first draft of the paper. DH and KR reviewed and edited the manuscript.

Declaration of interest statement

All authors declare no conflict of interest.

Patient and Public Involvement

Patients were not included in this modeling study.

Ethics approval

Not required.

Data sharing statement

All relevant data is included in the manuscript including in the supplementary materials.

References

1. Troeger C, Forouzanfar M, Rao PC, Khalil I, Brown A, Swartz S, et al. Estimates of the global, regional, and national morbidity, mortality, and aetiologies of lower respiratory tract infections in 195 countries: a systematic analysis for the Global Burden of Disease Study 2015. Lancet Infect Dis. 2017;17:1133-61. doi: 10.1016/S1473-3099(17)30396-1.

For peer review only - http://bmjopen.bmj.com/site/about/guidelines.xhtml

2. Shi T, McAllister DA, O'Brien KL, Simoes EAF, Madhi SA, Gessner BD, et al. Global, regional, and national disease burden estimates of acute lower respiratory infections due to respiratory syncytial virus in young children in 2015: a systematic review and modelling study. Lancet. 2017;390:946-58. doi: 10.1016/S0140-6736(17)30938-8.

3. Child Health and Mortality Prevention Surveillance (CHAMPS) [Internet]. c2020 [cited 2020 Apr 15]. Available from: https://champshealth.org/.

4. O'Brien KL, Baggett HC, Brooks WA, Feikin DR, Hammitt LL, Higdon MM, et al. Causes of severe pneumonia requiring hospital admission in children without HIV infection from Africa and Asia: the PERCH multi-country case-control study. Lancet. 2019;394:757-79. doi: 10.1016/S0140-6736(19)30721-4.

5. Utrecht University. News [Internet]. First consortium of local manufacturers to make affordable biosimilars available for low income countries. 9 Mar 2016. Available from: https://www.uu.nl/en/news/first-consortium-of-local-manufacturers-to-make-affordable-biosimilars-available-for-low-income.

6. Higgins D, Trujillo C, Keech C. Advances in RSV vaccine research and development – a global agenda. Vaccine. 2016;34:2870-5. doi: 10.1016/j.vaccine.2016.03.109.

7. PATH. RSV vaccine and mAb snapshot [Internet]. Seattle: PATH; March 2020 [cited 2019 Apr 23]. Available from: https://www.path.org/resources/rsv-vaccine-and-mab-snapshot/.

8. MedImmune LLC. A study to evaluate the safety and efficacy of MEDI8897 for the prevention of medically attended RSV LRTI in healthy preterm infants. (MEDI8897 Ph2b). 2017. Available from: https://www.clinicaltrials.gov/ct2/show/NCT02878330?term=NCT02878330&rank=1. [Cited 1 January 2017].

9. Gavi, the Vaccine Alliance. 06a - Annex C: Respiratory syncytial virus investment case. In: Vaccine Investment Strategy Programme and Policy Committee Meeting. 18-19 Oct 2018. Available from: https://www.gavi.org/sites/default/files/document/ppc-meeting-18-19-october-2018---vis-06a---annex-c--respiratory-syncytial-virus-investment-casepdf.pdf.

For peer review only - http://bmjopen.bmj.com/site/about/guidelines.xhtml

10. Ochalek J, Lomas J, Claxton K. Cost per DALY averted thresholds for low- and middle-income countries: evidence from cross country data. F1000Research. 2017;6:1–51. doi: 10.7490/f1000research.1113912.1.

11. Novavax, Inc. New data from Novavax Phase 3 Prepare™ trial of ResVax™ presented at 2019 IDSOG Annual Meeting [press release]. 12 Aug 2019. Available from: https://ir.novavax.com/news-releases/news-release-details/new-data-novavax-phase-3-preparetm-trial-resvaxtm-presented-0#.

12. Institute for Health Metrics and Evaluations (IHME) [Internet]. Global Burden of Disease Study 2017 (GBD 2017) Cause-Specific Mortality 1980-2017. c2019 [cited 2020 Jun 8]. Available from: http://ghdx.healthdata.org/record/ihme-data/gbd-2017-cause-specific-mortality-1980-2017.

13. Rudan I, O'Brien KL, Nair H, Liu L, Theodoratou E, Qazi S, et al. Epidemiology and etiology of childhood pneumonia in 2010: estimates of incidence, severe morbidity, mortality, underlying risk factors and causative pathogens for 192 countries. J Glob Health. 2013;3:10401. doi: 10.7189/jogh.03.010401.

14. Lawn JE, Blencowe H, Waiswa P, Amouzou A, Mathers C, Hogan D, et al. Stillbirths: rates, risk factors, and acceleration towards 2030. Lancet. 2016;387:587-603. doi: 10.1016/S0140-6736(15)00837-5.

15. U.S. Agency for International Development (USAID) [Internet]. Demographic and Health Surveys. c2020 [cited 2020 Jun 8]. Available from: https://www.dhsprogram.com/.

16. UNICEF [Internet]. Antenatal care. c2019 [cited 2020 Jun 8]. Available from: https://data.unicef.org/topic/maternal-health/antenatal-care/.

17. World Health Organization (WHO). WHO antenatal care randomized trial: manual for the implementation of the new model. Geneva: WHO; 2002. Available from: https://apps.who.int/iris/handle/10665/42513.

For peer review only

For peer review only - http://bmjopen.bmj.com/site/about/guidelines.xhtml

18. Baral R, Fleming J, Khan S, Higgins D, Hendrix N, Pecenka C. Inferring antenatal care visit timing in low- and middle-income countries: methods to inform potential maternal vaccine coverage. PLoS ONE. 2020;15(8): e0237718. https://doi.org/10.1371/journal.pone.0237718

19. World Health Organization (WHO) [Internet]. Data, statistics and graphics. c2020 [cited 2020 Jun 8]. Available from: https://www.who.int/immunization/monitoring_surveillance/data/en/.

20. Clark A, and Sanderson C. Timing of children's vaccinations in 45 low-income and middle-income countries: an analysis of survey data. Lancet. 2009. 373 (9674):1543–1549. 2008;23(8):579.

21. Debellut F, Clark A, Pecenka C, Tate J, Baral R, Sanderson C, Parashar U. Re-evaluating the potential impact and cost-effectiveness of rotavirus vaccination in 73 Gavi countries: a modeling study. Lancet Glob Health. 2019. 7: e1164–74.

22. World Health Organization (WHO). Preferred product characteristics for respiratory syncytial virus (RSV) vaccines. Geneva: WHO; 2017. Available from: http://www.who.int/immunization/documents/research/who_ivb_17.11/en/.

23. World Health Organization (WHO). Preferred product characteristics of monoclonal antibodies for passive immunization against respiratory syncytial virus (RSV). Geneva: WHO; 2020. Available from: https://www.who.int/immunization/research/ppc-tpp/PPC_RSV-MAbs_Draft_V-0.1-for-consultation.pdf?ua=1.

24. Gavi, the Vaccine Alliance [Internet]. Eligibility and transition policy. c2019 [cited 2019 May 20]. Available from: https://www.gavi.org/about/programme-policies/eligibility-and-transition/.

25. ImmunizationEconomics.org [Internet]. Immunization Costing Action Network (ICAN). c2020 [cited 2020 Jun 8]. Available from: http://immunizationeconomics.org/ican-home.

26. Wei C, Wang S, Yang Y, Al E, Li Ruili. Cost-effectiveness analysis of traditional Chinese medicine (TCM) and western medicine therapeutic schemes for 297 cases of child respiratory syncytial virus pneumonia. Chin J Pr Pediatr. 2008;23(8):579.

For peer review only - http://bmjopen.bmj.com/site/about/guidelines.xhtml

27. Xu H, Han X, Huang J. Multi-attribute evaluation of the clinical effect differences between Chinese medicine and western medicine in the treatment of pediatric RSV pneumonia. J Pediatr TCM. 2013;(1):23-27.

28. Zhang T, Zhu Q, Zhang X. Clinical characteristics and direct medical cost of respiratory syncytial virus infection in children hospitalized in Suzhou, China. Pediatr Infect Dis J. 2014;33(4):337-41. https://pubmed.ncbi.nlm.nih.gov/24088730/.

29. Chan PWK, Abdel-Latif MEA. Cost of hospitalization for respiratory syncytial virus chest infection and implications for passive immunization strategies in a developing nation. Acta Paediatr. 2003;92:481-5. doi: 10.1111/j.1651-2227.2003.tb00582.x.

30. Bhuiyan MU, Luby SP, Alamgir NI, Homaira N, Sturm-Ramirez K, Gurley ES, et al. Costs of hospitalization with respiratory syncytial virus illness among children aged <5 years and the financial impact on households in Bangladesh, 2010. J Glob Health. 2017;7:10412. doi: 10.7189/jogh.07.010412.

31. Khuri-Bulos N, Williams JV, Shehabi AA, Faouri S, Al Jundi E, Abushariah O, et al. Burden of respiratory syncytial virus in hospitalized infants and young children in Amman, Jordan. Scand J Infect Dis. 2010;42:368-74. doi: 10.3109/00365540903496544.

32. Zhang S, Sammon PM, King I, Andrade AL, Toscano CM, Araujo SN, et al. Cost of management of severe pneumonia in young children: systematic analysis. J Glob Health. 2016;6:10408. https://pubmed.ncbi.nlm.nih.gov/27231544.

33. Institute for Health Metrics and Evaluations (IHME) [Internet]. Global Burden of Disease Study 2010 (GBD 2010) Disability Weights. c2019 [cited 2020 Jun 8]. Available from: http://ghdx.healthdata.org/record/ihme-data/gbd-2010-disability-weights.

34. Graham BS, Anderson LJ. Challenges and opportunities for respiratory syncytial virus vaccines. Curr Top Microbiol Immunol. 2013;372:391-404. doi: 10.1007/978-3-642-38919-1_20.

For peer review only - http://bmjopen.bmj.com/site/about/guidelines.xhtml

35. World Health Organization (WHO) [Internet]. Children Aged <5 Years with Pneumonia Symptoms Taken to a Healthcare Provider (%) (Child Health). c2017 [cited 2020 Jun 8]. Available from: https://apps.who.int/gho/data/node.imr.WHS4_106?lang=en.

36. Li X, Willem L, Antillon M, Bilcke J, Jit M, Beutels P. Health and economic burden of respiratory syncytial virus (RSV) disease and the cost-effectiveness of potential interventions against RSV among children under 5 years in 72 Gavi-eligible countries. BMC Med. 2020;18:82. doi: 10.1186/s12916-020-01537-6.

37. Williams AL et al. RSV-associated respiratory death among Zambian infants. Poster presentation at: RSV18. 2018.

38. Caballero MT, Bianchi AM, Nuño A, Ferretti AJP, Polack LM, Remondino I, et al. Mortality associated with acute respiratory infections among children at home. J Infect Dis. 2019;219:358-64. doi: 10.1093/infdis/jiy517.

39. Bertram MY, Lauer JA, De Joncheere K, Edejer T, Hutubessy R, Kieny MP, et al. Cost-effectiveness thresholds: pros and cons. Bull World Health Organ. 2016;94:925-30. doi: 10.2471/BLT.15.164418.

40. Cromer D, van Hoek AJ, Newall AT, Pollard AJ, Jit M. Burden of paediatric respiratory syncytial virus disease and potential effect of different immunisation strategies: a modelling and cost-effectiveness analysis for England. Lancet Public Health. 2017;2:e367-74. doi: 10.1016/S2468-2667(17)30103-2.

41. United Nations Department of Economic and Social Affairs, Population Division [Internet]. World Population Prospects 2019. c2019 [cited 2019 Dec 15]. Available from: https://population.un.org/wpp/.

For peer review only

For peer review only - http://bmjopen.bmj.com/site/about/guidelines.xhtml

Tables

Table 1. Input parameter values used for modeling.

<table>
<tr><th>Input</th><th>RSV maternal vaccine</th><th>RSV monoclonal antibody</th><th>Sources</th></tr>
<tr><td colspan="4">Intervention specific inputs</td></tr>
<tr><td>Target population</td><td>126 million
(annual average number of pregnant women, between 2030 and 2039, across 131 countries)</td><td>124 million
(annual average live births between 2030 and 2039, across 131 countries)</td><td>Birth estimates and population growth rate [41]; still birth rates [14]</td></tr>
<tr><td>Intervention schedule</td><td>Single dose vaccine given during weeks 24–36 of gestation, as a part of ANC</td><td>Single dose mAb given to newborn at birth</td><td>[22]; expert opinion</td></tr>
<tr><td>Efficacy against RSV endpoints</td><td>Baseline: cases = 40.9%; hospitalization = 41.7%; death = 59.6%
Minimum scenario: 30% (for all endpoints)
Maximum scenario: 90% (for all endpoints)</td><td>Baseline: cases = 60%; hospitalization = 60%; death = 70%
Minimum scenario: 30% (for all endpoints)
Maximum scenario: 90% (for all endpoints)</td><td>[11]; [22,23], expert opinion</td></tr>
<tr><td>Duration of protection against RSV+</td><td>Baseline: 3 months
Minimum scenario: 4 months
Maximum scenario: 6 months</td><td>Baseline: 6 months
Minimum scenario: 4 months
Maximum scenario: 6 months</td><td>[22,23] and expert opinion</td></tr>
<tr><td>Efficacy against all cause LRTI*</td><td>Cases = 25%; hospitalization = 25%; death = 39%</td><td>Cases = 25%; hospitalization = 25%; death = 39%</td><td>[11]; expert opinion</td></tr>
<tr><td>Duration of protection against all cause LRTI*</td><td>6 months</td><td>6 months</td><td>[11]; expert opinion</td></tr>
<tr><td>Intervention coverage</td><td>Derived from ANC coverage (average 84%, range: 40% to 96%, in 2030)</td><td>Derived from BCG coverage (average 82%, range: 48% to 98%, in 2030)</td><td>[15,17,19]</td></tr>
<tr><td colspan="4">Common to both interventions</td></tr>
<tr><td colspan="4">Disease burden</td></tr>
<tr><td>Incidence of RSV ALRI</td><td colspan="2">Country-specific incidence for 0–5 years for envelope (35.3 to 65.6)

Developing-country estimate by narrow age band for case distribution by age;<table><tr><td colspan="2">Annual incidence per 1,000 children</td></tr><tr><td>0–27 days</td><td>40.0</td></tr><tr><td>28–< 3 months</td><td>45.7</td></tr><tr><td>3–5 months</td><td>99.6</td></tr><tr><td>6–11 months</td><td>98.8</td></tr><tr><td>12–23 months</td><td>79.1</td></tr></table>
Rescaled to match country-specific incidence envelope</td><td>[2]</td></tr>
<tr><td>Incidence of severe RSV ALRI</td><td colspan="2">Developing-country estimates with uniform age distribution<table><tr><td colspan="2">Annual incidence of severe RSV ALRI per 1,000 children</td></tr><tr><td>0–5 months</td><td>36.1</td></tr><tr><td>6–11 months</td><td>24.7</td></tr><tr><td>0–59 months</td><td>10.2</td></tr></table></td><td>[2]</td></tr>
</table>

For peer review only - http://bmjopen.bmj.com/site/about/guidelines.xhtml

Hospital admissions for RSV-associated ALRI	Annual hospital admissions for RSV-associated ALRI per 1,000 children<br>0–5 months: 20.2<br>6–11 months: 11	[2]
Hospital case fatality	Hospital case fatality risk (%), by age group<br>0–5 months: 2.2<br>6–11 months: 2.4	[2]
RSV-ALRI mortality	Hospital deaths *2.2 (*adjusted for community deaths*) *0.9 (*adjusted for influenza activities*)	[2]
Incidence of all-cause LRTI	Country-specific;<br>By ages: early neonates (0 –7 days), post neonates (7–28 days), late neonates (1–12 months); Burden for post neonates, uniformly distributed across ages by month	[12]
Incidence of severe LRTI	11.5% of all incidence resulting in severe cases	Assumed (based on the estimates used in [13]
Hospital admissions for LRTI	40% of all severe cases resulting in hospital admissions	Assumed
Mortality due to LRTI	Country specific, early neonates, post neonates, late neonates; burden for post neonates uniformly distributed across ages by month	[12]
Age distribution of LRTI	Assumes uniform distribution of burden across months by age	Assumed
**Costs**		
Intervention cost	$3 per dose in Gavi countries; $5 per dose in non-Gavi countries	Assumed
Intervention delivery costs	Mean incremental economic cost of delivery per dose: $0.63 in LICs; $1.73 in LMICs and UMICs	[25]
Treatment cost	Cost of managing severe pneumonia in LMICs (outpatients $53; inpatients $250)	[32]
Vaccine introduction dates	National introduction starting 2030	Product development timeline, assumed
**Other assumptions**		
DALY weights	Severe ALRI = 0.21; Non-severe ALRI = 0.053	[33]
Duration of illness	Severe ALRI = 10 days; Non-severe ALRI = 5 days	[34]
Length of hospital stay	Length of stay for severe pneumonia in LMICs, 6.4 days	[32]
Health care seeking	Health seeking for children with pneumonia, country specific	[35]

Abbreviations: ANC, antenatal care; BCG, Bacillus Calmette–Guérin; LIC, low-income country; LMIC, low- and middle-income country; LRTI, lower respiratory tract infection; mAb, monoclonal antibody; UMIC, upper-middle-income country; RSV, respiratory syncytial virus; WHO, World Health Organization; ALRI, acute lower respiratory illness; DALY, disability-adjusted life year; LMIC, low- and middle-income country; LRTI, lower respiratory tract infection; RSV, respiratory syncytial virus.

[+]Duration of protection in the minimum scenario is higher than in the baseline scenario. For maternal vaccine baseline, we assume duration of protection data from a recent clinical trial that failed to meet the primary endpoint. Nonetheless, in anticipation that a successful product would likely have higher duration of protection than three months, we evaluate the minimum scenario at four months duration of protection.

*Used in adjunct scenario only. The adjunct scenario evaluates intervention impact on all-cause LRTI mortality.

For peer review only - http://bmjopen.bmj.com/site/about/guidelines.xhtml

Table 2. Summary of disease burden, impact and cost effectiveness ratios, with and without intervention (2030–2039), baseline scenario.

Country group by	N	Disease burden without intervention				Burden averted and ICER with RSV maternal vaccine					Burden averted and ICER with RSV monoclonal antibody				
		Non-severe cases	Severe cases	Hospitalizations	Deaths	Non-severe cases	Severe cases	Hospitalizations	Deaths	ICER per DALY averted	Non-severe cases	Severe cases	Hospitalizations	Deaths	ICER per DALY averted
Gavi status															
Gavi	73	31,288,677	10,683,106	8,031,827	352,990	2,159,630	1,730,164	1,333,545	83,024	1,073	13,866,799	4,742,022	3,565,171	182,800	315
Non-Gavi	58	10,657,947	4,599,391	3,457,938	151,973	819,749	907,088	699,149	43,528	1,681	5,610,598	2,441,921	1,835,898	94,133	577
World Bank income group															
LIC	34	10,823,869	3,562,172	2,678,130	117,701	760,348	577,452	445,078	27,710	949	4,774,083	1,573,199	1,182,771	60,645	257
LMIC	46	22,502,889	7,872,029	5,918,389	260,107	1,559,549	1,292,990	996,588	62,046	1,311	10,083,892	3,536,660	2,658,950	136,334	428
UMIC	51	8,619,867	3,848,296	2,893,246	127,155	659,482	766,809	591,027	36,796	1,631	4,619,422	2,074,084	1,559,348	79,954	551
WHO geographic region															
EAP	20	5,313,235	3,097,972	2,329,133	102,363	314,036	582,849	449,238	27,969	1,411	2,570,416	1,558,912	1,172,029	60,094	479
ECA	20	2,609,512	633,363	476,178	20,928	244,307	125,417	96,666	6,018	1,425	1,398,536	337,329	253,612	13,004	437
LAC	23	2,583,464	1,024,279	770,079	33,844	209,296	204,837	157,881	9,829	1,507	1,349,631	536,105	403,057	20,666	507
MENA	13	2,907,732	1,058,521	795,823	34,976	222,211	192,789	148,594	9,251	1,566	1,468,837	535,629	402,699	20,648	532
SA	8	12,207,891	4,018,853	3,021,474	132,791	842,810	643,028	495,622	30,857	1,138	5,628,427	1,857,416	1,396,452	71,601	342
SSA	47	16,324,790	5,449,509	4,097,078	180,062	1,146,719	888,332	684,693	42,628	1,169	7,061,551	2,358,554	1,773,220	90,920	359
Total	131	41,946,624	15,282,497	11,489,765	504,963	2,979,379	2,637,252	2,032,693	126,552	1,342	19,477,397	7,183,943	5,401,069	276,933	431

For peer review only - http://bmjopen.bmj.com/site/about/guidelines.xhtml

Abbreviations: LIC, low-income country; LMIC, low- and middle-income country; RSV, respiratory syncytial virus; UMIC, upper-middle-income country; WHO, World Health Organization; EAP: East Asia & Pacific; ECA: Europe & Central Asia; LAC: Latin America & Caribbean; MENA: Middle East & North Africa; SA: South Asia; SSA: Sub-Saharan Africa.

For peer review only

Figures

Figure 1. Incremental cost-effectiveness ratios as a percentage of national GDP per capita.

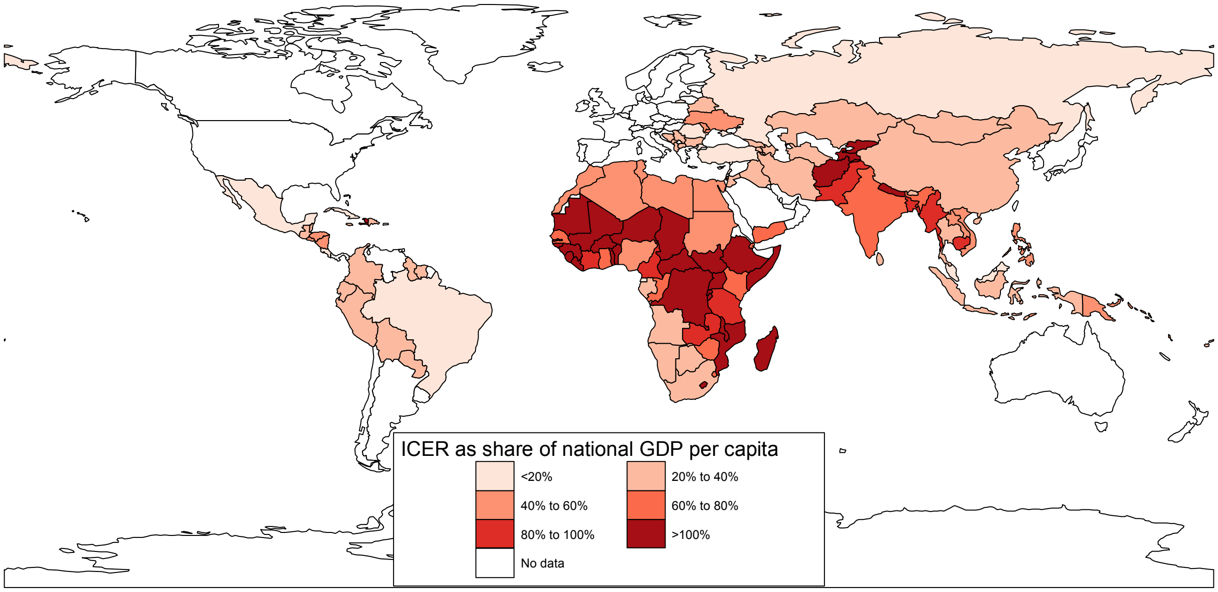

Panel a. Maternal vaccine

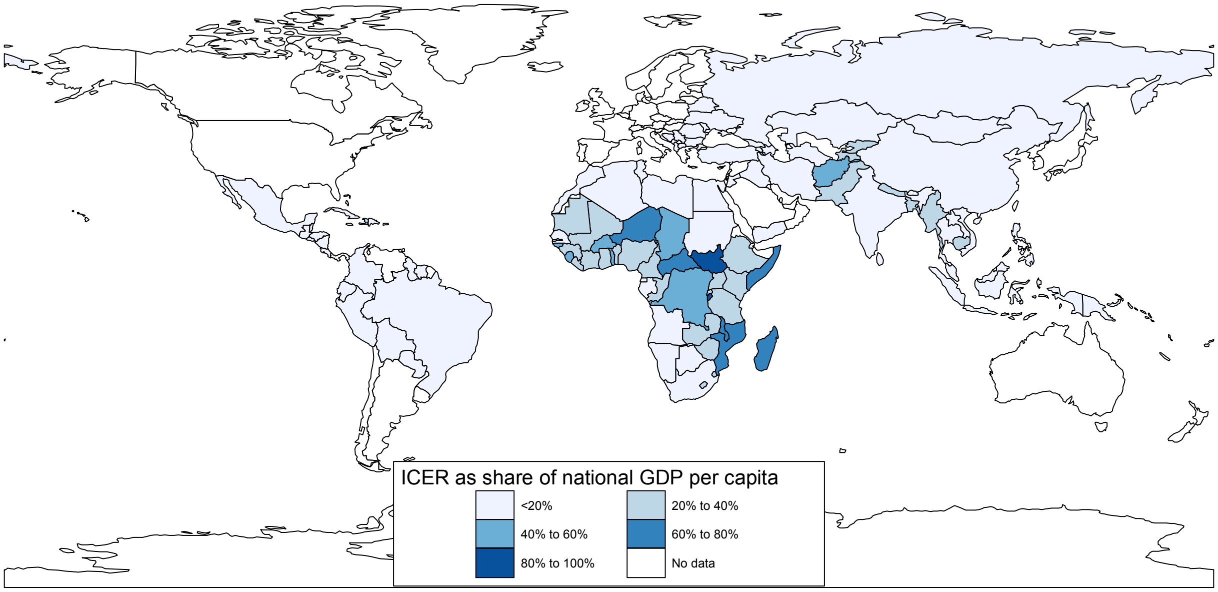

Panel b. Monoclonal antibody

For peer review only - http://bmjopen.bmj.com/site/about/guidelines.xhtml

Figure 2. Impact of change in key input parameter values on deaths averted.

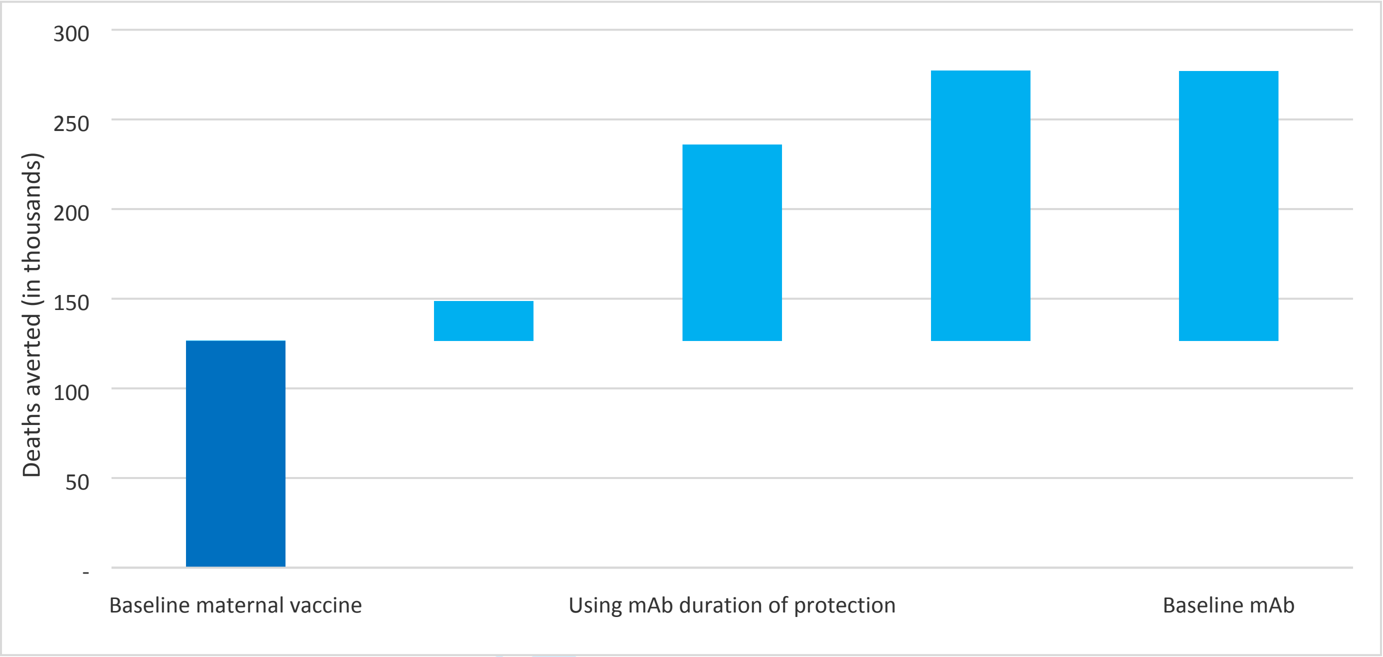

For peer review only - http://bmjopen.bmj.com/site/about/guidelines.xhtml

Figure 3. Average incremental cost-effectiveness ratios by country groups.

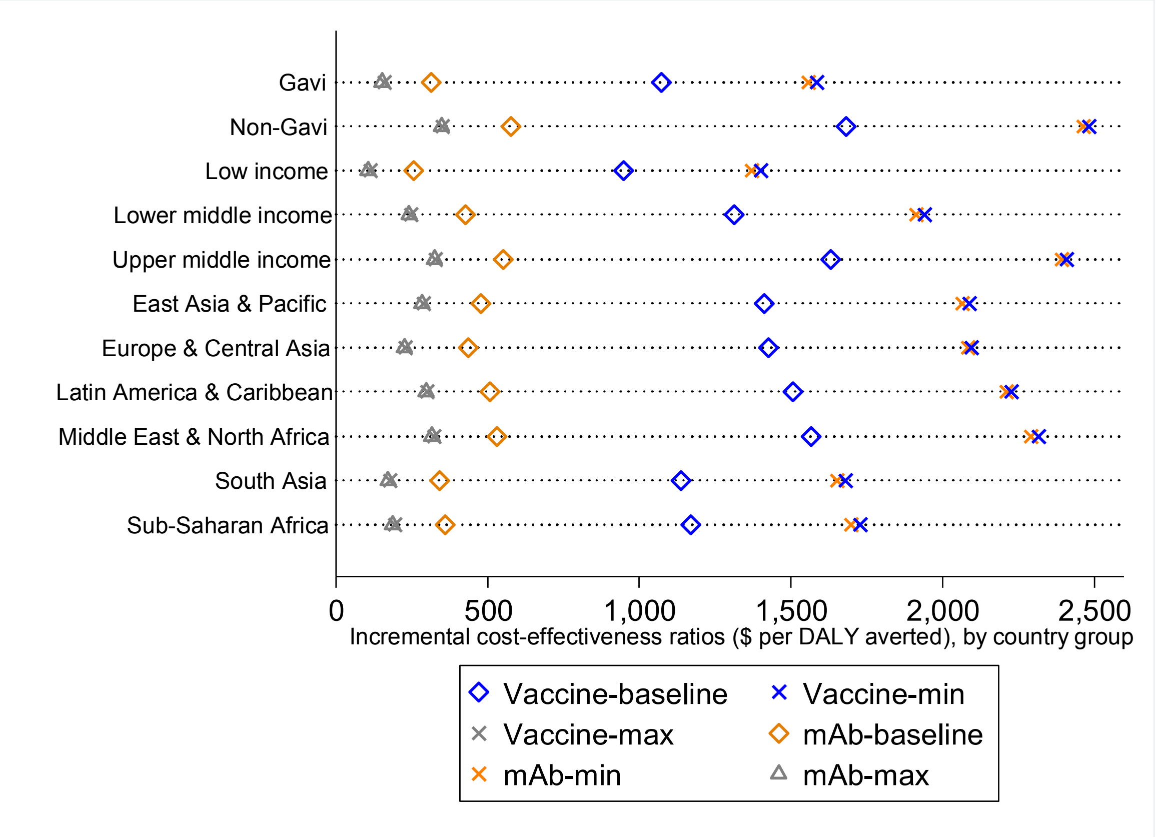

For peer review only - http://bmjopen.bmj.com/site/about/guidelines.xhtml

Figure captions

Figure 1. Incremental cost-effectiveness ratios as a percentage of national GDP per capita.

Figure 2. Impact of change in key input parameter values on deaths averted.

Figure 3. Average incremental cost-effectiveness ratios by country groups.

For peer review only

For peer review only - http://bmjopen.bmj.com/site/about/guidelines.xhtml

Figure 1. Incremental cost-effectiveness ratios as a percentage of national GDP per capita.

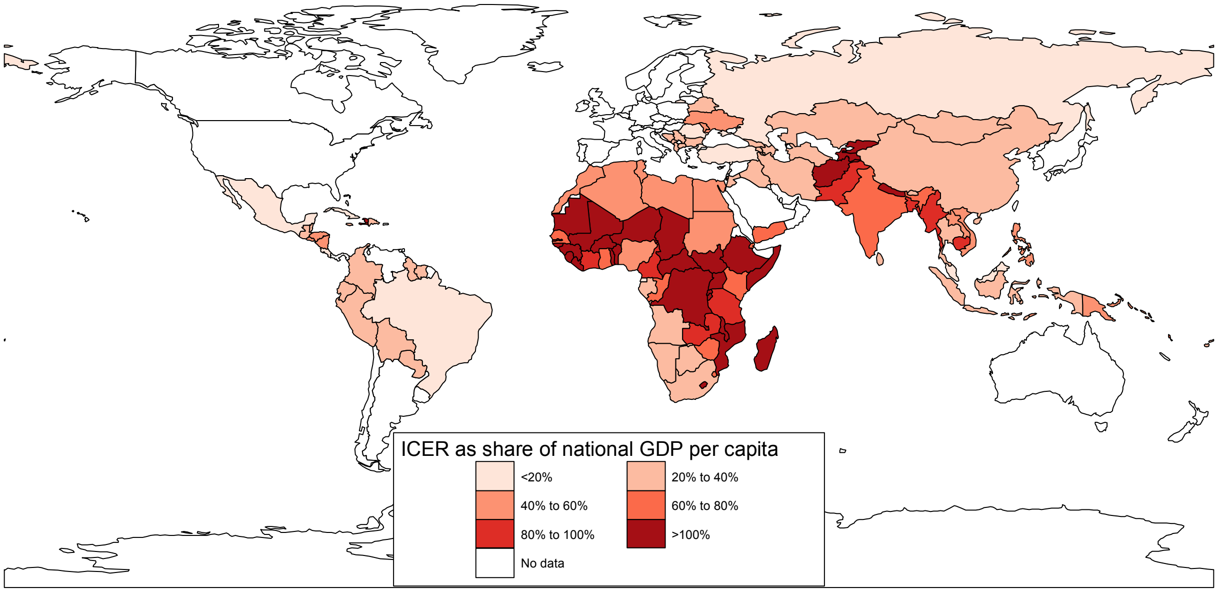

Panel a. Maternal vaccine

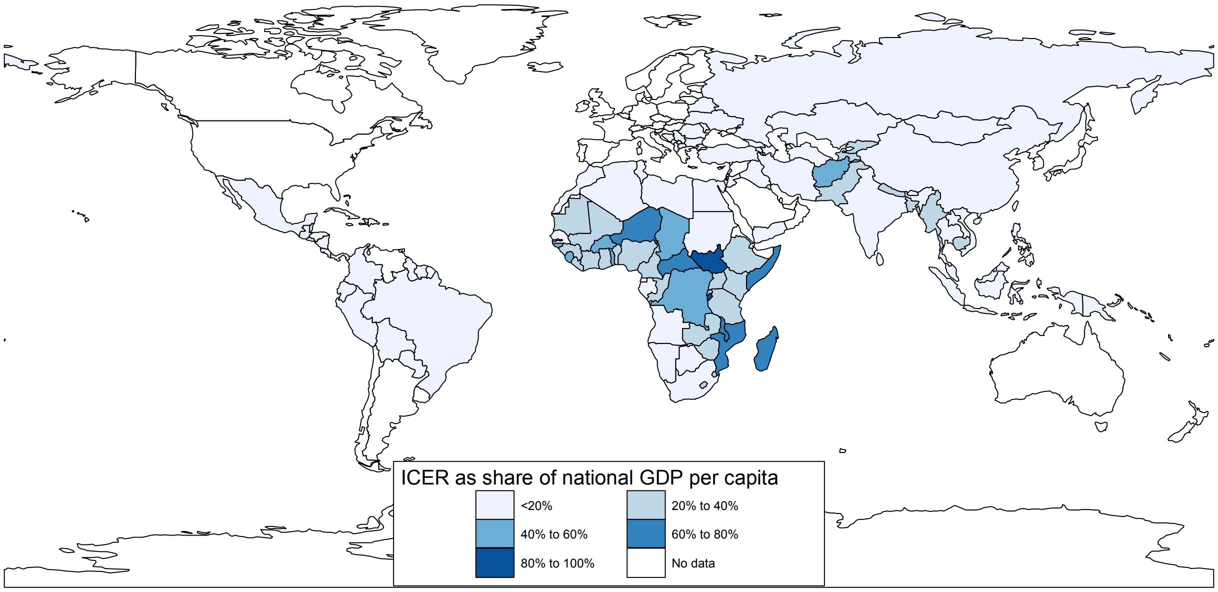

Panel b. Monoclonal antibody

Figure 2. Impact of change in key input parameter values on deaths averted.

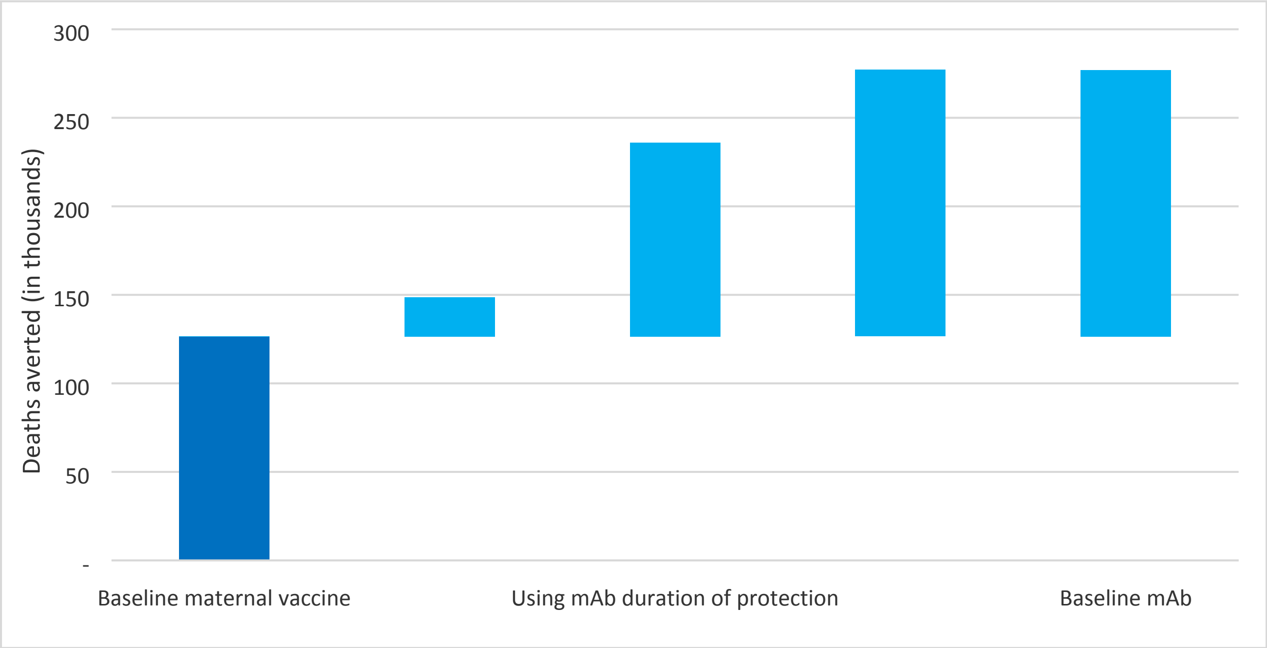

For peer review only

Figure 3. Average incremental cost-effectiveness ratios by country groups.

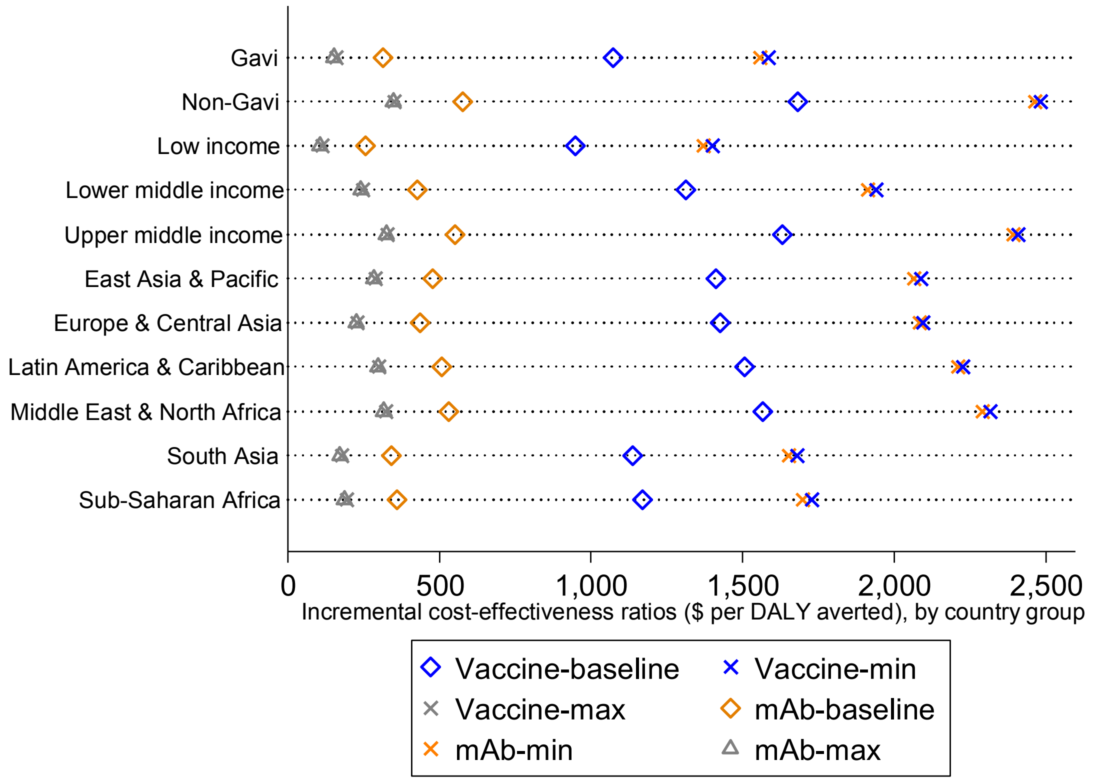

Appendix Table 1. Cost of intervention and incremental cost-effectiveness ratios by country group (2030–2039).

Country group by	Maternal vaccine			Monoclonal antibody		
	Health care cost averted	Cost of vaccination program	ICER per DALY averted	Health care cost averted	Cost of vaccination program	ICER per DALY averted
Gavi status						
Gavi (N=73)	397,985,940	3,065,106,927	1,073	1,311,275,193	3,028,242,872	315
Non-Gavi (N=58)	204,118,914	2,398,496,379	1,681	663,227,127	2,355,785,836	577
World Bank Country income group						
LIC (N=34)	131,178,798	886,636,675	949	420,421,498	867,885,189	257
LMIC (N=46)	299,612,949	2,557,430,903	1,311	995,875,548	2,522,516,388	428
UMIC (N=51)	171,313,107	2,019,535,727	1,631	558,205,274	1,993,627,132	551
Geographic region						
East Asia & Pacific (N=20)	124,384,911	1,411,293,535	1,411	392,896,831	1,380,338,857	479
Europe & Central Asia (N=20)	32,220,016	301,556,780	1,425	109,878,013	295,554,675	437
Latin America & Caribbean (N=23)	46,562,615	515,567,727	1,507	146,732,622	492,924,565	507
Middle East & North Africa (N=13)	45,216,714	496,642,134	1,566	154,303,356	504,915,613	532
South Asia (N=8)	155,092,982	1,187,286,456	1,138	558,355,660	1,236,166,379	342
Sub-Saharan Africa (N=47)	198,627,617	1,551,256,674	1,169	612,335,839	1,474,128,619	359
Total (N=131)	602,104,854	5,463,603,306	1,342	1,974,502,320	5,384,028,709	431

For peer review only

Abbreviations: DALY, disability-adjusted life year; ICER, incremental cost-effectiveness ratio; LIC, low-income country; LMIC, low- and middle-income country; UMIC, upper-middle-income country.

Appendix Table 2. Incremental cost-effectiveness ratio per disability-adjusted life year (DALY) averted as a percentage of gross domestic product (GDP) per capita across various scenarios.

		Maternal vaccine						Monoclonal antibody					
Scenarios		Baseline	Baseline low price	Baseline high price	Minimum	Maximum	Adjunct	Baseline	Baseline low price	Baseline high price	Minimum	Maximum	Adjunct
Efficacy (%)		Trial (40%–60%)	Trial (40%–60%)	Trial (40%–60%)	30	90	Trial (25%–39%)	60%–70%	60%–70%	60%–70%	30	90	Trial (25%–39%)
Duration of Protection (months)		3	3	3	4	6	6	6	6	6	4	6	6
Coverage (%)		1	1	1	1	1	1	4	4	4	4	4	4
Intervention cost (Gavi/non-Gavi)		$3/$5	$1.5/$2.5	$6/$10	$3/$5	$3/$5	$3/$5	$3/$5	$1.5/$2.5	$6/$10	$3/$5	$3/$5	$3/$5
Afghanistan	1	163%	82%	325%	240%	16%	7%	41%	5%	113%	234%	15%	7%
Albania		37%	21%	69%	55%	6%	17%	11%	4%	26%	55%	6%	17%
Algeria		43%	25%	79%	64%	10%	35%	15%	7%	31%	63%	9%	35%
Angola	1	36%	23%	63%	54%	7%	4%	12%	6%	24%	53%	7%	4%
Armenia	1	31%	20%	55%	47%	6%	14%	10%	5%	21%	47%	6%	14%
Azerbaijan	1	30%	19%	52%	44%	5%	2%	9%	4%	19%	44%	5%	2%
Bangladesh	1	83%	52%	145%	123%	15%	8%	27%	13%	55%	122%	15%	8%
Belarus		32%	18%	59%	46%	4%	105%	9%	3%	21%	46%	4%	104%
Belize		34%	20%	63%	51%	7%	12%	12%	5%	25%	50%	7%	12%
Benin	1	122%	63%	240%	181%	20%	11%	37%	11%	90%	177%	19%	11%
Bhutan	1	40%	25%	70%	59%	7%	8%	12%	6%	26%	58%	7%	8%
Bolivia	1	36%	23%	63%	54%	6%	7%	11%	5%	23%	53%	6%	7%
Bosnia & Herzegovina		33%	19%	61%	48%	5%	156%	10%	3%	23%	48%	5%	156%

Botswana		26%	15%	47%	39%	7%	10%	10%	5%	19%	38%	7%	10%
Brazil		19%	11%	35%	28%	4%	11%	7%	3%	14%	28%	4%	11%
Bulgaria		22%	13%	40%	32%	4%	19%	7%	3%	15%	32%	4%	18%
Burkina Faso	1	158%	80%	315%	233%	17%	10%	43%	7%	114%	232%	17%	10%
Burundi	1	333%	168%	663%	490%	33%	27%	86%	12%	235%	484%	31%	27%
Cambodia	1	90%	56%	157%	133%	14%	7%	27%	12%	58%	132%	14%	7%
Cameroon	1	93%	59%	162%	139%	19%	11%	31%	16%	61%	134%	18%	11%
Cape Verde		56%	33%	102%	83%	13%	25%	20%	9%	40%	81%	12%	24%
C.A. Rep.	1	260%	133%	513%	386%	40%	9%	76%	20%	188%	373%	37%	9%
Chad	1	148%	76%	291%	220%	25%	7%	44%	13%	107%	211%	22%	7%
China		21%	12%	38%	31%	5%	13%	8%	4%	15%	31%	5%	13%
Colombia		28%	16%	51%	41%	6%	16%	10%	5%	21%	41%	6%	16%
Comoros	1	75%	38%	148%	111%	10%	9%	20%	4%	52%	105%	9%	9%
Congo	1	71%	45%	123%	106%	15%	14%	25%	13%	49%	106%	15%	14%
Costa Rica		14%	8%	26%	21%	3%	27%	5%	2%	10%	21%	3%	27%
Cuba	1	13%	8%	23%	18%	0%	22%	2%	0%	7%	19%	0%	22%
Côte d'Ivoire	1	86%	55%	150%	128%	16%	7%	28%	14%	57%	126%	16%	7%
N. Korea	1												
D. Rep. Congo	1	212%	109%	419%	314%	30%	14%	59%	14%	150%	301%	27%	13%
Djibouti	1	61%	38%	108%	90%	7%	11%	16%	5%	37%	88%	6%	11%
Dominican Republic		25%	14%	45%	37%	5%	8%	9%	4%	18%	36%	5%	8%
Ecuador		27%	16%	49%	40%	6%	10%	10%	5%	20%	40%	6%	10%
Egypt		50%	29%	92%	74%	11%	8%	18%	8%	37%	74%	11%	8%
El Salvador		44%	26%	81%	66%	9%	22%	15%	7%	32%	64%	9%	21%
Equatorial Guinea		20%	12%	37%	30%	5%	4%	7%	4%	15%	30%	5%	4%
Eritrea	1												
Ethiopia	1	127%	65%	251%	188%	18%	15%	37%	9%	93%	185%	17%	15%
Fiji		34%	20%	63%	51%	8%	9%	12%	6%	25%	50%	8%	9%
Gabon		26%	15%	47%	38%	5%	5%	9%	4%	18%	37%	5%	5%
Gambia	1	130%	65%	260%	191%	10%	19%	32%	3%	91%	190%	10%	18%
Georgia	1	30%	19%	53%	45%	5%	42%	9%	4%	20%	44%	5%	41%
Ghana	1	63%	40%	110%	94%	12%	11%	21%	10%	42%	92%	12%	11%

Grenada		17%	10%	31%	25%	4%	8%	6%	3%	12%	25%	4%	8%
Guatemala		40%	24%	74%	60%	9%	5%	15%	7%	30%	60%	9%	5%
Guinea	1	134%	68%	265%	199%	20%	8%	40%	10%	99%	197%	19%	8%
Guinea-Bissau	1	147%	75%	291%	218%	21%	20%	43%	10%	108%	215%	20%	19%
Guyana	1	25%	16%	44%	37%	3%	10%	7%	3%	15%	36%	3%	9%
Haiti	1	123%	62%	246%	181%	10%	8%	28%	1%	83%	174%	8%	8%
Honduras	1	47%	29%	82%	69%	7%	44%	14%	6%	30%	68%	7%	43%
India	1	65%	41%	114%	96%	9%	6%	19%	7%	41%	95%	8%	6%
Indonesia	1	32%	20%	57%	48%	5%	8%	10%	4%	21%	48%	5%	8%
Iran		32%	19%	59%	47%	7%	29%	11%	5%	23%	47%	7%	29%
Iraq		38%	22%	70%	56%	8%	18%	13%	6%	27%	55%	8%	18%
Jamaica		34%	20%	63%	51%	7%	36%	12%	5%	25%	50%	7%	35%
Jordan		41%	24%	74%	60%	8%	20%	13%	6%	29%	59%	8%	19%
Kazakhstan		22%	13%	41%	32%	4%	10%	7%	2%	15%	32%	3%	10%
Kenya	1	81%	51%	141%	120%	13%	8%	25%	11%	52%	118%	12%	8%
Kiribati	1	76%	48%	132%	113%	13%	23%	24%	12%	50%	112%	13%	23%
Kyrgyzstan	1	106%	67%	184%	157%	20%	34%	35%	17%	70%	156%	20%	33%
Lao PDR	1	51%	32%	88%	76%	10%	3%	16%	8%	33%	73%	9%	2%
Lebanon		19%	11%	34%	27%	4%	54%	6%	3%	13%	27%	4%	53%
Lesotho	1	121%	76%	211%	179%	19%	10%	36%	16%	76%	173%	18%	10%
Liberia	1	130%	66%	258%	192%	15%	17%	35%	7%	93%	189%	15%	16%
Libya		40%	24%	74%	60%	8%	109%	14%	6%	29%	59%	8%	108%
Madagascar	1	234%	120%	462%	346%	33%	14%	66%	16%	167%	335%	30%	14%
Malawi	1	307%	154%	614%	449%	21%	36%	70%	1%	207%	438%	18%	35%
Malaysia		17%	10%	32%	26%	4%	27%	6%	3%	13%	26%	4%	27%
Maldives		16%	9%	29%	23%	4%	52%	6%	3%	12%	23%	4%	52%
Mali	1	125%	65%	247%	186%	21%	12%	39%	11%	93%	183%	20%	12%
Mauritania	1	117%	74%	203%	174%	24%	15%	38%	19%	76%	167%	22%	15%
Mauritius		17%	10%	31%	25%	4%	18%	6%	3%	13%	25%	4%	18%
Mexico		20%	11%	36%	29%	4%	11%	7%	3%	14%	29%	4%	11%
Micronesia		55%	32%	100%	81%	13%	23%	20%	10%	40%	81%	13%	23%
Mongolia	1	33%	21%	57%	48%	6%	4%	11%	5%	21%	48%	6%	4%
Montenegro		23%	13%	42%	33%	3%	108%	7%	2%	15%	33%	3%	108%

Morocco		58%	34%	106%	85%	12%	24%	20%	9%	41%	84%	12%	24%
Mozambique	1	245%	124%	487%	361%	28%	34%	67%	12%	177%	360%	27%	34%
Myanmar	1	98%	62%	169%	144%	18%	9%	32%	16%	64%	144%	18%	9%
Namibia		39%	23%	72%	58%	9%	7%	14%	6%	29%	58%	9%	7%
Nepal	1	109%	54%	221%	160%	3%	11%	23%	-2%	73%	159%	3%	11%
Nicaragua	1	52%	33%	91%	77%	8%	15%	16%	7%	33%	76%	8%	15%
Niger	1	266%	135%	529%	392%	29%	20%	68%	10%	185%	379%	26%	19%
Nigeria	1	63%	40%	109%	93%	14%	3%	21%	11%	42%	91%	13%	3%
Pakistan	1	86%	54%	151%	127%	13%	9%	24%	10%	52%	120%	11%	8%
Papua New Guinea	1	51%	33%	89%	76%	10%	8%	17%	8%	34%	75%	10%	8%
Paraguay		31%	18%	57%	46%	6%	23%	10%	5%	22%	45%	6%	23%
Peru		27%	16%	50%	40%	6%	9%	10%	5%	20%	40%	6%	9%
Philippines		58%	34%	106%	85%	12%	10%	20%	9%	42%	85%	12%	10%
Republic of Moldova	1	58%	37%	102%	86%	9%	12%	18%	8%	38%	86%	9%	12%
Romania		17%	10%	31%	25%	3%	7%	5%	2%	12%	25%	3%	7%
Russian Federation		19%	11%	35%	28%	3%	25%	6%	2%	13%	28%	3%	25%
Rwanda	1	127%	65%	252%	188%	15%	16%	34%	6%	90%	183%	14%	15%
Saint Lucia		18%	10%	33%	26%	4%	13%	6%	3%	13%	26%	4%	13%
St. Vincent & Grenad.		23%	14%	43%	35%	5%	11%	8%	4%	17%	35%	5%	11%
Samoa		45%	26%	81%	66%	11%	26%	16%	8%	33%	65%	11%	26%
S. Tome & Principe	1	68%	43%	118%	100%	11%	13%	20%	9%	43%	98%	10%	13%
Senegal	1	73%	37%	146%	108%	7%	10%	19%	3%	52%	106%	7%	10%
Serbia		29%	17%	55%	43%	4%	114%	8%	3%	20%	43%	4%	113%
Sierra Leone	1	194%	97%	388%	285%	15%	11%	47%	3%	134%	282%	14%	10%
Solomon Islands	1	60%	38%	105%	89%	11%	15%	19%	9%	38%	86%	10%	15%
Somalia	1	217%	113%	425%	323%	41%	17%	69%	23%	161%	310%	38%	16%
South Africa		33%	19%	61%	49%	7%	8%	11%	5%	24%	48%	7%	7%
South Sudan	1	396%	196%	797%	580%	20%	14%	85%	-4%	264%	564%	17%	14%
Sri Lanka	1	30%	19%	52%	44%	6%	67%	10%	5%	20%	44%	6%	66%

State of Palestine		59%	35%	108%	89%	16%	124%	23%	12%	45%	88%	16%	123%
Sudan	1	51%	32%	89%	75%	9%	9%	16%	8%	33%	74%	9%	9%
Suriname		30%	18%	55%	44%	7%	10%	11%	5%	22%	44%	6%	10%
Swaziland		64%	38%	117%	95%	14%	7%	23%	11%	47%	95%	14%	7%
Syria													
Macedonia		31%	18%	57%	45%	4%	45%	9%	3%	21%	45%	4%	44%
Tajikistan	1	116%	60%	230%	172%	16%	7%	34%	8%	85%	171%	16%	7%
Thailand		28%	16%	51%	41%	6%	32%	10%	5%	20%	41%	6%	32%
Timor-Leste	1	60%	38%	104%	89%	11%	7%	19%	9%	38%	86%	10%	7%
Togo	1	162%	82%	321%	239%	19%	22%	44%	8%	115%	234%	18%	21%
Tonga		47%	28%	86%	70%	11%	24%	17%	8%	35%	69%	11%	23%
Tunisia		46%	27%	84%	68%	10%	83%	16%	8%	33%	67%	10%	83%
Turkey		15%	9%	27%	22%	3%	25%	5%	2%	11%	22%	3%	25%
Turkmenistan		27%	16%	50%	40%	5%	4%	9%	4%	19%	40%	5%	4%
Uganda	1	157%	78%	315%	230%	9%	29%	35%	0%	105%	224%	8%	29%
Ukraine	1	52%	33%	90%	76%	8%	89%	16%	7%	33%	76%	8%	89%
Tanzania	1	103%	52%	203%	151%	12%	10%	27%	5%	72%	146%	11%	10%
Uzbekistan	1	56%	35%	97%	83%	10%	5%	18%	9%	37%	82%	10%	5%
Vanuatu		62%	37%	113%	92%	15%	10%	23%	11%	46%	91%	15%	10%
Venezuela													
Viet Nam	1	51%	32%	89%	75%	9%	21%	16%	8%	34%	75%	9%	21%
Yemen	1	69%	35%	137%	102%	10%	13%	20%	5%	50%	100%	9%	13%
Zambia	1	99%	62%	173%	146%	15%	11%	29%	13%	62%	143%	14%	11%
Zimbabwe	1	74%	37%	146%	109%	9%	5%	21%	4%	54%	108%	9%	5%

Note: Country by Gavi status (Gavi country = 1). Current GDP values were not available for 4 countries (Venezuela, Syria, Eretria, North Korea) and were excluded from the analysis.

*Negative cost-effectiveness ratio implies cost savings.

Cells in green indicate lower ICER to GDP ratio and cells in red indicate higher ICER to GDP ratio.

Additional file 1

EVEREST Statement: Checklist for health economics paper

	Study section	Additional remarks
**Study design**		
(1) The research question is stated	Introduction	
(2) The economic importance of the research question is stated	Introduction	
(3) The viewpoint(s) of the analysis are clearly stated and justified	Methods; Discussion	
(4) The rationale for choosing the alternative programmes or interventions compared is stated	Introduction; Methods	
(5) The alternatives being compared are clearly described	Methods	
(6) The form of economic evaluation used is stated	Introduction; Methods	
(7) The choice of form of economic evaluation is justified in relation to the questions addressed	Introduction; Discussion	
**Data collection**		
(8) The source(s) of effectiveness estimates used are stated	Methods-Assumptions used in the model; Table 1	
(9) Details of the design and results of effectiveness study are given (if based on single study)	N/A	Data derived from peer reviewed literature, Target product profile
(10) Details of the method of synthesis or meta-analysis of estimates are given (if based on an overview of a number of effectiveness studies)	N/A	
(11) The primary outcome measure(s) for the economic evaluation are clearly stated	Methods	
(12) Methods to value health states and other benefits are stated	N/A	
(13) Details of the subjects from whom valuations were obtained are given	N/A	
(14) Productivity changes (if included) are reported separately	N/A	
(15) The relevance of productivity changes to the study question is discussed	N/A	
(16) Quantities of resources are reported separately from their unit costs	Methods-Assumptions used in the model; Table 1	

For peer review only

(17) Methods for the estimation of quantities and unit costs are described	Methods-Assumptions used in the model; Table 1	
(18) Currency and price data are recorded	Methods-Cost calculations; Tables 1- 5	
(19) Details of currency of price adjustments for inflation or currency conversion are given	N/A	As the study is looking for relative cost
(20) Details of any model used are given	Methods	
(21) The choice of model used and the key parameters on which it is based are justified	Methods	
**Analysis and interpretation of results**		
(22) Time horizon of costs and benefits is stated	Methods	
(23) The discount rate(s) is stated	N/A	
(24) The choice of rate(s) is justified	N/A	
(25) An explanation is given if costs or benefits are not discounted	N/A	
(26) Details of statistical tests and confidence intervals are given for stochastic data	N/A	
(27) The approach to sensitivity analysis is given	Methods-Sensitivity analysis	
(28) The choice of variables for sensitivity analysis is justified	Methods; Discussion	
(29) The ranges over which the variables are varied are stated	Table 1	
(30) Relevant alternatives are compared	Methods	
(31) Incremental analysis is reported	Methods; Table 2; Figures 1, 2, 3	
(32) Major outcomes are presented in a disaggregated as well as aggregated form	Table 2; Appendix Tables 1 and 2	
(33) The answer to the study question is given	Results, Discussion; Conclusion	
(34) Conclusions follow from the data reported	Discussion; Conclusion	
(35) Conclusions are accompanied by the appropriate caveats	Discussion; Conclusion	

For peer review only - http://bmjopen.bmj.com/site/about/guidelines.xhtml

BMJ Open

Impact and cost-effectiveness of potential interventions against infant respiratory syncytial virus (RSV) in 131 low- and middle-income countries using a static cohort model

Journal:	*BMJ Open*
Manuscript ID	bmjopen-2020-046563.R1
Article Type:	Original research
Date Submitted by the Author:	18-Mar-2021
Complete List of Authors:	Baral, Ranju; Program for Appropriate Technology in Health, Center for Vaccine Innovation and Access<br>Higgins , Deborah ; Program for Appropriate Technology in Health, Center for Vaccine Innovation and Access<br>Regan, Katie; Program for Appropriate Technology in Health, Center for Vaccine Innovation and Access<br>Pecenka, Clint; Program for Appropriate Technology in Health, Center for Vaccine Innovation and Access
**Primary Subject Heading**:	Health economics
Secondary Subject Heading:	Health economics, Respiratory medicine
Keywords:	Health economics < HEALTH SERVICES ADMINISTRATION & MANAGEMENT, PAEDIATRICS, Respiratory infections < THORACIC MEDICINE

SCHOLARONE™
Manuscripts

I, the Submitting Author has the right to grant and does grant on behalf of all authors of the Work (as defined in the below author licence), an exclusive licence and/or a non-exclusive licence for contributions from authors who are: i) UK Crown employees; ii) where BMJ has agreed a CC-BY licence shall apply, and/or iii) in accordance with the terms applicable for US Federal Government officers or employees acting as part of their official duties; on a worldwide, perpetual, irrevocable, royalty-free basis to BMJ Publishing Group Ltd ("BMJ") its licensees and where the relevant Journal is co-owned by BMJ to the co-owners of the Journal, to publish the Work in this journal and any other BMJ products and to exploit all rights, as set out in our licence.

The Submitting Author accepts and understands that any supply made under these terms is made by BMJ to the Submitting Author unless you are acting as an employee on behalf of your employer or a postgraduate student of an affiliated institution which is paying any applicable article publishing charge ("APC") for Open Access articles. Where the Submitting Author wishes to make the Work available on an Open Access basis (and intends to pay the relevant APC), the terms of reuse of such Open Access shall be governed by a Creative Commons licence – details of these licences and which Creative Commons licence will apply to this Work are set out in our licence referred to above.

Other than as permitted in any relevant BMJ Author's Self Archiving Policies, I confirm this Work has not been accepted for publication elsewhere, is not being considered for publication elsewhere and does not duplicate material already published. I confirm all authors consent to publication of this Work and authorise the granting of this licence.

For peer review only

Impact and cost-effectiveness of potential interventions against infant respiratory syncytial virus (RSV) in 131 low- and middle-income countries using a static cohort model

Authors:

Ranju Baral, Center for Vaccine Innovation and Access, PATH, Seattle, USA

Deborah Higgins, Center for Vaccine Innovation and Access, PATH, Seattle, USA

Katie Regan, Center for Vaccine Innovation and Access, PATH, Seattle, USA

Clint Pecenka, Center for Vaccine Innovation and Access, PATH, Seattle, USA

Corresponding author:

Ranju Baral, Health Economist

Center for Vaccine Innovation and Access, PATH, Seattle, USA

rbaral@path.org

206-285-3500

Running title:

Infant RSV interventions in LMIC

For peer review only

For peer review only - http://bmjopen.bmj.com/site/about/guidelines.xhtml

Abstract

Objectives. Interventions to prevent childhood respiratory syncytial virus (RSV) disease are limited and costly. New interventions are in advanced stages of development and could be available soon. This study aims to evaluate the potential impact and cost-effectiveness of two interventions to prevent childhood RSV —a maternal vaccine and a monoclonal antibody (mAb).

Design. Using a static population-based cohort model, we evaluate impact and cost-effectiveness of RSV interventions, from a health systems perspective. The assumed baseline efficacy and duration of protection were higher for the mAb (60-70% efficacy, protection 6 months) compared to the maternal vaccine (40-60% efficacy, protection 3 months). Both interventions were evaluated at US$3 and $5 per dose for Gavi and non-Gavi countries, respectively. A range of input values were considered to explore uncertainty.

Settings. 131 low- and middle-income countries.

Participants. Pregnant women and live birth cohorts.

Interventions. Maternal vaccine given to pregnant women and monoclonal antibody given to young infants.

Primary and secondary outcome measures. Disability adjusted life years averted, severe case averted, deaths averted, incremental cost effectiveness ratios.

Results. Under baseline assumptions, maternal vaccine and mAbs were projected to avert 25% and 55% of RSV-related deaths among infants younger than six months of age, respectively. The average incremental cost-effectiveness ratio per disability-adjusted life year averted was $1,342 (range $800 to $1,866) for maternal RSV vaccine and $431 (range $167 to $692) for mAbs. At a 50% gross domestic product per capita threshold, maternal vaccine and mAbs were cost-effective in 60 and 118 countries, respectively.

Conclusions. Both interventions are projected to be impactful and cost-effective in many countries, a finding that would be enhanced if country-specific Gavi co-financing to eligible countries were included.

For peer review only - http://bmjopen.bmj.com/site/about/guidelines.xhtml

mAbs, with assumed higher efficacy and duration of protection, are expected to be more cost-effective than RSV maternal vaccines at similar prices. Final product characteristics will influence this finding.

Key words: Respiratory syncytial virus (RSV); maternal RSV vaccine; RSV monoclonal antibody; health impact; cost-effectiveness.

Strengths and limitations of this study

- This is one of the first studies to examine the potential impact and cost-effectiveness of maternal vaccines and monoclonal antibodies for RSV prevention, across 131 low-and middle-income countries.
- This study compares products with uncertain characteristics using the latest available data on vaccine characteristics, supplemented by the target product profile to inform the model parameters.
- A range of input values were considered to explore uncertainty, insights from which are useful to inform subsequent intervention development.
- Final product characteristics and product prices will determine the relative cost-effectiveness of RSV interventions.

For peer review only

Introduction

Respiratory syncytial virus (RSV) is a common cause of acute lower respiratory illness (ALRI) among children younger than age five, causing between 41,000 and 118,000 child deaths per year globally [1,2]. RSV disease is most severe among young infants, and the burden is highest in low- and middle-income countries (LMICs), where more than 99% of RSV deaths occur [2]. Emerging evidence indicates the unrecognized burden of RSV among children in low-resource settings is also significant, with up to 10% of young infant deaths attributable to RSV infection [3,4].

Existing RSV interventions are limited and cost prohibitive, even in high-income countries [5]. Several prophylactic interventions are currently under development [6,7]. Multiple maternal vaccine candidates designed to protect against RSV illness in infants are in relatively advanced stages of development and expected to be available for global use in the coming years [6]. Monoclonal antibodies (mAbs) are available and in use for high-risk babies in high-income countries. However, the available mAbs are not only expensive but require multiple doses during the RSV season. Long lasting more affordable mAbs that are easier to deliver in low resource settings are in advanced stages of development [8]. Given the extent of the global RSV disease burden—especially in low-income countries (LICs)—and the lack of efficacious and cost-effective therapeutic options, these new interventions are expected to be included in Gavi, the Vaccine Alliance's, portfolio [9], subject to licensure, prequalification, and cost characteristics. In this paper, we estimate the potential impact and cost-effectiveness of a maternal vaccine and a mAb, both designed to avert RSV disease burden in young infants in LMICs. We compare each intervention against a scenario of no intervention and against each other. Results from this study illustrate the potential benefits of these products and will help inform decisions around further development. This analysis will also inform global and LMIC decision-makers likely to face choices about whether and which interventions to introduce.

Methods

For peer review only - http://bmjopen.bmj.com/site/about/guidelines.xhtml

We examined the potential impact and cost-effectiveness of a single-dose RSV maternal vaccine administered to pregnant women at 24 to 36 weeks gestation, and of a single-dose mAb given to infants directly at birth across 131 LMICs, compared to no intervention. Both interventions were evaluated independently using a static cohort model. For maternal vaccine, infants born two weeks following maternal immunization were considered as protected to allow time for immune transfer from mother to children. All children receiving mAbs were considered protected immediately.

We examined the impact and cost of interventions from the health systems perspective over the period 2030 to 2039 (10 years), assuming nationwide introduction in 2030. Primary input values for a baseline scenario are given in Table 1. Key model outputs include cases averted, severe cases averted, hospitalizations averted, deaths averted, disability adjusted life years (DALYs) averted, and the incremental cost per DALY averted due to RSV interventions. Given the inconsistent use of country-specific cost-effectiveness thresholds across LMICs, we used a willingness-to-pay threshold of 0.5 times the gross domestic product (GDP) per capita in each country [10]. Results are summarized by World Health Organization (WHO) regions, World Bank income group, and Gavi eligibility to understand impact by country group. All monetary units are adjusted to 2016 US dollars.

Table 1. Key input parameter values used for modeling.

**Input**	**RSV maternal vaccine**	**RSV monoclonal antibody**	**Sources**
Intervention specific inputs			
Target population	126 million<br>(annual average number of pregnant women, between 2030 and 2039, across 131 countries)	124 million<br>(annual average live births between 2030 and 2039, across 131 countries)	Birth estimates and population growth rate [11]; still birth rates [12]
Intervention schedule	Single dose vaccine given during weeks 24–36 of gestation, as a part of ANC	Single dose mAb given to newborn at birth	[13, 14]; expert opinion
Efficacy against RSV endpoints	Baseline: cases = 40.9%; hospitalization = 41.7%; death = 59.6%<br>Minimum scenario: 30% (for all endpoints)<br>Maximum scenario: 90% (for all endpoints)	Baseline: cases = 60%; hospitalization = 60%; death = 70%<br>Minimum scenario: 30% (for all endpoints)<br>Maximum scenario: 90% (for all endpoints)	[13,15,16], expert opinion
Duration of protection against RSV^{+}	Baseline: 3 months<br>Minimum scenario: 4 months<br>Maximum scenario: 6 months	Baseline: 6 months<br>Minimum scenario: 4 months<br>Maximum scenario: 6 months	[13, 16] and expert opinion
Efficacy against all cause LRTI*	Cases = 25%; hospitalization = 25%; death = 39%	Cases = 25%; hospitalization = 25%; death = 39%	[15]; expert opinion
Duration of protection against all cause LRTI*	6 months	6 months	[15]; expert opinion

For peer review only - http://bmjopen.bmj.com/site/about/guidelines.xhtml

For peer review only

Intervention coverage	Derived from ANC coverage (average 84%, range: 40% to 96%, in 2030)	Derived from BCG coverage (average 82%, range: 48% to 98%, in 2030)	[17, 18, 19, 20]
Common to both interventions			
Disease burden			
Incidence of RSV ALRI	Country-specific incidence for 0–5 years for envelope (35.3 to 65.6).<br>Developing-country estimate by narrow age band for case distribution by age;<br>Annual incidence per 1,000 children<br>0–27 days 40.0<br>28–< 3 months 45.7<br>3–5 months 99.6<br>6–11 months 98.8<br>12–23 months 79.1<br>*Rescaled to match country-specific incidence envelope.*		[2]
Incidence of severe RSV ALRI	Developing-country estimates with uniform age distribution<br>Annual incidence of severe RSV ALRI per 1,000 children<br>0–5 months 36.1<br>6–11 months 24.7<br>0–59 months 10.2		[2]
Hospital admissions for RSV-associated ALRI	Annual hospital admissions for RSV-associated ALRI per 1,000 children<br>0–5 months 20.2<br>6–11 months 11		[2]
Hospital case fatality	Hospital case fatality risk (%), by age group<br>0–5 months 2.2<br>6–11 months 2.4		[2]
RSV-ALRI mortality	Hospital deaths *2.2 (*adjusted for community deaths*) *0.9 (*adjusted for influenza activities*)		[2]
Incidence of all-cause LRTI	Country-specific;<br>By ages: early neonates (0 –7 days), post neonates (7–28 days), late neonates (1–12 months); Burden for post neonates, uniformly distributed across ages by month		[21]
Incidence of severe LRTI	11.5% of all incidence resulting in severe cases		Assumed (based on the estimates used in [22])
Hospital admissions for LRTI	40% of all severe cases resulting in hospital admissions		Assumed
Mortality due to LRTI	Country specific, early neonates, post neonates, late neonates; burden for post neonates uniformly distributed across ages by month		[21]
Age distribution of LRTI	Assumes uniform distribution of burden across months by age		Assumed
Costs			
Intervention cost	$3 per dose in Gavi countries; $5 per dose in non-Gavi countries		Assumed
Intervention delivery costs	Mean incremental economic cost of delivery per dose: $0.63 in LICs; $1.73 in LMICs and UMICs		[23]
Treatment cost	Cost of managing severe pneumonia in LMICs (outpatients $53; inpatients $250)		[24]
Vaccine introduction dates	National introduction starting 2030		Product development timeline, assumed
Other assumptions			
DALY weights	Severe ALRI = 0.21; Non-severe ALRI = 0.053		[25]
Duration of illness	Severe ALRI = 10 days; Non-severe ALRI = 5 days		[26]
Length of hospital stay	Length of stay for severe pneumonia in LMICs, 6.4 days		[24]
Health care seeking	Health seeking for children with pneumonia, country specific		[27]

Abbreviations: ANC, antenatal care; BCG, Bacillus Calmette–Guérin; LIC, low-income country; LMIC, low- and middle-income country; LRTI, lower respiratory tract infection; mAb, monoclonal antibody; UMIC, upper-middle-income country; RSV, respiratory syncytial virus; WHO, World Health Organization; ALRI, acute lower respiratory illness; DALY, disability-adjusted life year; LMIC, low- and middle-income country; LRTI, lower respiratory tract infection; RSV, respiratory syncytial virus.

[+]Duration of protection in the minimum scenario is higher than in the baseline scenario. For maternal vaccine baseline, we assume duration of protection data from a recent clinical trial that failed to meet the primary endpoint. Nonetheless, in

For peer review only - http://bmjopen.bmj.com/site/about/guidelines.xhtml

anticipation that a successful product would likely have higher duration of protection than three months, we evaluate the minimum scenario at four months duration of protection.
*Used in adjunct scenario only. The adjunct scenario evaluates intervention impact on all-cause LRTI mortality.

Disease burden

Disease burden inputs including disease incidence, severe disease incidence, incidence of hospitalizations, and mortality were derived from a comprehensive systematic review paper [2]. We combined country-specific disease incidence estimates in children under five years of age with a representative developing-country estimate to generate incidence by granular age band in each country. To generate incidence of severe disease, hospitalization, and hospital mortality, we used developing-country estimates [2]. Estimated hospital deaths were adjusted by multiplying by 1.98 to account for community deaths and influenza coinfection [2]. The actual values of disease burden inputs are given in Table 1.

Some RSV interventions under development have shown promising results in their ability to avert all-cause lower respiratory tract infections (LRTIs) among children [15], in addition to RSV infection. Thus, we also explored the potential impact of both RSV interventions on all-cause LRTI, based on emerging burden data, using estimates from the Global Burden of Disease Study 2017 [21], and assuming a uniform distribution of disease among children one to 12 months of age. Further, we assumed 11.5% of all-cause LRTI cases would result in severe cases [22] and 40% of all severe cases would result in hospitalization.

Intervention introduction and coverage

The leading RSV intervention candidates could be available for use in the next five-to-eight years [7]. We assumed both interventions would be available by 2030 and all countries would begin national introduction in 2030.

All pregnant women attending antenatal care (ANC) visits were assumed eligible to receive RSV maternal vaccine. To project the number of pregnant women per country, we added country-specific stillbirths [12] to the United Nations Population Division annual birth projections [11]. We estimated maternal vaccine coverage during the 24- to 36-week vaccination window by examining country-specific ANC first-visit timing [17], country-specific ANC coverage [18], and the WHO's recommended ANC

For peer review only - http://bmjopen.bmj.com/site/about/guidelines.xhtml

timing based on the focused ANC model guideline [19]. Details on methods used in estimating maternal vaccine coverage during ANC within a specific gestation window is described elsewhere [28].

All live births were considered eligible for mAbs. All infants were assumed to be covered at the Bacillus Calmette–Guérin (BCG) vaccine birth dose coverage levels adjusted to the timeliness of vaccine receipt. Country's overall BCG coverage were derived from the most recent WHO/UNICEF estimates of national immunization coverage [20]. Timeliness of BCG birth dose receipt was derived using the methods described in the literature [29,30].

Coverage levels for both interventions for each country are projected to improve by 3 percentage points each year until coverage reaches 70%, and after that by 1 percentage point each year until reaching 95% coverage. This projection was made to correspond with methods applied during the Gavi vaccine investment strategy [9].

Intervention characteristics

Our analysis assumed a single dose maternal RSV vaccine would be given to pregnant women between 24 and 36 months of gestation, inferred based on the WHO preferred product characteristics (PPCs) [13] and other ongoing clinical trials [14]. We based vaccine efficacy and duration of protection on data from one of the first maternal vaccine candidate Phase 3 clinical trials (Table 1) [15]. Other maternal vaccines are in clinical development which may have improved efficacy. Given the uncertainty in vaccine characteristics, scenario analyses included a range in efficacy (30% to 90%) and duration of protection afforded to infants (three to six months).

Our analysis assumed a single dose mAb would be given to newborns at birth, would have 60% to 70% efficacy, and would offer protection for six months, inferred based on the PPCs [16] and other studies [31, 32]. As with the maternal vaccine, we varied efficacy and duration of protection in scenario analysis. We assumed neither intervention contributed to herd immunity, and that efficacy did not wane during the period of protection.

Intervention price and delivery costs

For peer review only

For both interventions, we assumed a per-dose price of US$3 in Gavi-eligible countries and per-dose price of $5 in LMICs not eligible for Gavi support. Traditionally mAbs are more expensive to produce than a vaccine and will likely have higher market price than a vaccine. If Gavi decides to support RSV interventions once they are available, Gavi-eligible countries would likely be able to access the interventions at varying prices depending on their transition status [33]. We refrained from projecting individual country Gavi eligibility or intervention prices due to significant uncertainty, and instead evaluated a range of intervention prices in sensitivity analyses.

Given the paucity of data on maternal immunization and mAb delivery costs in LMICs, we used delivery costs estimates for other vaccines derived from the Immunization Costing Action Network (ICAN) repository [23]. Unit costs of delivering RSV interventions were $0.63 for LICs and $1.73 for LMICs. We accounted for vaccine/mAbs wastage at 5% and a buffer stock at 25% of demand in the introduction year, and at 25% of the incremental demand in subsequent years.

Health service costs

Very few studies have analyzed the cost of managing RSV in children, especially in LMICs [34–39]. Hospitalization costs also vary widely. In Bangladesh, for example, hospitalization averages $74, whereas in China it averages $662. Given limited RSV-specific information in LMICs, we used the average cost of treating pneumonia in young children, identified in a systematic review [24] as $53.26 and $250.04 per outpatient and inpatient episode, respectively. We assumed that severe cases seek inpatient care and non-severe cases seek outpatient care.

Cost-effectiveness analysis

We calculated intervention costs by multiplying the number of doses (estimated number of pregnant women receiving vaccine for maternal vaccine and estimated number of live births for mAbs) with the unit cost of delivery and cost per dose. We estimated averted health care costs by multiplying the estimated number of non-severe/severe cases averted by the costs of an outpatient/inpatient episode. Vaccine impact was calculated by multiplying the respective disease burden in children born two weeks after maternal vaccination with vaccine efficacy. The mAb impact was calculated by multiplying disease

For peer review only

For peer review only - http://bmjopen.bmj.com/site/about/guidelines.xhtml

burden with the BCG coverage estimates and mAb efficacy. We estimated health outcomes including severe/non-severe cases averted, hospitalizations averted, deaths averted, and DALYs averted for each country and year. Disability weights for non-severe and severe ALRI were used to compute DALYs [25]. Further, we assumed duration of illness at five days for non-severe disease and 10 days for severe disease [26]. The length of a hospital stay for severe disease was assumed to be 6.4 days [24]. Both undiscounted and discounted DALYs (at 3% discount rate) were generated for the analysis. We also accounted for variation in health-seeking practices by using health care use data from children younger than five years receiving pneumonia care [27].

We calculated incremental cost-effectiveness ratios (ICERs) for each country by dividing the net cost of intervention by the net DALYs averted by the intervention.

Sensitivity analysis

We conducted one-way sensitivity analysis by changing the values of key input parameters, including intervention efficacy, duration of protection, anticipated coverage, and intervention price. Alternate scenarios that changed one or more input parameters to evaluate results sensitivity were also considered. In an adjunct scenario, we evaluated how different interventions show impact on all-cause LRTI mortality, using the efficacy and duration of protection values as suggested by recent clinical trial data [15], and disease burden for all-cause LRTI from the 2017 Global Burden of Disease Study [21].

Patient and Public Involvement

Patients were not included in this modeling study.

Results

Disease burden without interventions

Over the 10-year period, about 41.94 million non-severe cases, 15.28 million severe cases, 11.48 million hospitalizations, and 504,963 thousand deaths among children younger than six months of age in 131

For peer review only - http://bmjopen.bmj.com/site/about/guidelines.xhtml

LMICs are projected (Table 2). Seventy-three Gavi-eligible countries accounted for 70% of the mortality burden. Most deaths would occur in sub-Saharan Africa (36%, 47 countries), followed by South Asia (26%, eight countries).

For peer review only

For peer review only - http://bmjopen.bmj.com/site/about/guidelines.xhtml

Table 2. Summary of disease burden, impact and cost effectiveness ratios, with and without intervention (2030–2039), baseline scenario.

Country group by	N	Disease burden without intervention				Burden averted and ICER with RSV maternal vaccine					Burden averted and ICER with RSV monoclonal antibody				
		Non-severe cases	Severe cases	Hospitalizations	Deaths	Non-severe cases	Severe cases	Hospitalizations	Deaths	ICER per DALY averted	Non-severe cases	Severe cases	Hospitalizations	Deaths	ICER per DALY averted
Gavi status															
Gavi	73	31,288,677	10,683,106	8,031,827	352,990	2,159,630	1,730,164	1,333,545	83,024	1,073	13,866,799	4,742,022	3,565,171	182,800	315
Non-Gavi	58	10,657,947	4,599,391	3,457,938	151,973	819,749	907,088	699,149	43,528	1,681	5,610,598	2,441,921	1,835,898	94,133	577
World Bank income group															
LIC	34	10,823,869	3,562,172	2,678,130	117,701	760,348	577,452	445,078	27,710	949	4,774,083	1,573,199	1,182,771	60,645	257
LMIC	46	22,502,889	7,872,029	5,918,389	260,107	1,559,549	1,292,990	996,588	62,046	1,311	10,083,892	3,536,660	2,658,950	136,334	428
UMIC	51	8,619,867	3,848,296	2,893,246	127,155	659,482	766,809	591,027	36,796	1,631	4,619,422	2,074,084	1,559,348	79,954	551
WHO geographic region															
EAP	20	5,313,235	3,097,972	2,329,133	102,363	314,036	582,849	449,238	27,969	1,411	2,570,416	1,558,912	1,172,029	60,094	479
ECA	20	2,609,512	633,363	476,178	20,928	244,307	125,417	96,666	6,018	1,425	1,398,536	337,329	253,612	13,004	437
LAC	23	2,583,464	1,024,279	770,079	33,844	209,296	204,837	157,881	9,829	1,507	1,349,631	536,105	403,057	20,666	507
MENA	13	2,907,732	1,058,521	795,823	34,976	222,211	192,789	148,594	9,251	1,566	1,468,837	535,629	402,699	20,648	532
SA	8	12,207,891	4,018,853	3,021,474	132,791	842,810	643,028	495,622	30,857	1,138	5,628,427	1,857,416	1,396,452	71,601	342
SSA	47	16,324,790	5,449,509	4,097,078	180,062	1,146,719	888,332	684,693	42,628	1,169	7,061,551	2,358,554	1,773,220	90,920	359
Total	131	41,946,624	15,282,497	11,489,765	504,963	2,979,379	2,637,252	2,032,693	126,552	1,342	19,477,397	7,183,943	5,401,069	276,933	431

For peer review only - http://bmjopen.bmj.com/site/about/guidelines.xhtml

Abbreviations: LIC, low-income country; LMIC, low- and middle-income country; RSV, respiratory syncytial virus; UMIC, upper-middle-income country; WHO, World Health Organization; EAP: East Asia & Pacific; ECA: Europe & Central Asia; LAC: Latin America & Caribbean; MENA: Middle East & North Africa; SA: South Asia; SSA: Sub-Saharan Africa.

For peer review only

For peer review only - http://bmjopen.bmj.com/site/about/guidelines.xhtml

Expected health outcomes with intervention

RSV maternal vaccine, under the baseline scenario, has the potential to avert 2.97 million non-severe cases, 2.63 million severe cases, 2.03 million hospitalizations, 126,552 deaths, and 3.73 million DALYs (discounted) among children younger than six months of age across all countries over 10 years (Table 2). Globally, about 17% of severe RSV cases and 25% of RSV-related deaths among infants under six months of age would be averted by RSV maternal vaccine, which is roughly 13 deaths averted per 100,000 vaccinated pregnant women.

An RSV mAb, under the baseline scenario, is expected to avert 19.47 million cases of non-severe disease, 7.18 million severe cases, 5.40 million hospitalizations, 276,933 deaths, and 8.19 million DALYs (discounted) among children younger than six months of age across all countries over 10 years (Table 2). Globally, about 55% of RSV deaths among infants younger than six months of age would be averted with RSV mAbs—equivalent to approximately 28 averted deaths per 100,000 newborns receiving the intervention.

Under alternative scenarios using varying efficacy and duration of protection assumptions (minimum and maximum scenarios), the RSV maternal vaccine is estimated to avert between 84,934 and 356,346 deaths over 10 years; and the RSV mAb is expected to avert roughly 84, 864 and 356, 057 deaths. Assuming both interventions are able to affect all-cause LRTI, as suggested by recent clinical trial data [15], either intervention is projected to avert roughly 1.05 million LRTI deaths (29% of all LRTI deaths) among children younger than six months of age in LMICs.

Cost-effectiveness of interventions

The average annual cost of vaccination programs across all countries for the duration of analysis was estimated to be about $546.36 million and $538.40 million for RSV maternal vaccine and mAbs, respectively. The economic benefits expressed in terms of cost-of-care averted was about $602.10 million (maternal vaccine) and $1.97 billion (mAbs) over the 10 years (see appendix Table 1).

For maternal RSV vaccine, the ICER per DALY averted is estimated at $1,342 ($1,073 across Gavi-eligible countries and $1,681 across non-Gavi countries). Similarly, the ICER estimates for RSV mAbs is

For peer review only

For peer review only - http://bmjopen.bmj.com/site/about/guidelines.xhtml

$431 ($315 across Gavi-eligible countries and $577 across non-Gavi countries). It is important to note these ICERs reflect the full potential cost of either intervention. Countries eligible for Gavi support would be expected to pay a share of the prices used in this analysis, thus reducing the ICER from the country perspective.

Results from alternative scenarios with low and high efficacy and duration of protection assumptions show that costs per DALY averted across countries range from $244 to $1,982 (maternal vaccine) and $239 to $1,958 (mAbs). By reducing the intervention price to 50% of the baseline price (i.e., $1.50 for Gavi-eligible countries and $2.50 for non-Gavi countries), the average ICER per DALY averted would decline to $781 (range $45 to $1,147) for the maternal vaccine and $178 (range $42 to $1,132) for the mAb. Increasing the intervention price by 200% of the baseline price, the average ICER per DALY averted increases to $2,465 (range $642 to $3,651) for the maternal vaccine and $938 (range $632 to $3,610) for the mAbs.

When comparing ICERs against an individual country's income level at baseline, the maternal vaccine ICERs were less than 50% of the GDP per capita in 60 countries (12 Gavi and 48 non-Gavi), suggesting intervention cost-effectiveness in those countries. ICERs for RSV mAbs were below the 50% GDP per capita threshold in 118 countries (62 Gavi-eligible and all non-Gavi). For both interventions, countries with higher ICER to GDP per capita ratios are concentrated in sub-Saharan Africa and Asia (Figures 1 and 2). Many of these countries remain eligible for Gavi support and are expected to pay lower intervention prices. As a result, the cost per DALY averted from the perspective of these countries is likely to be much more favorable than shown here. For example, if each of the original Gavi-eligible countries were responsible for half of the cost of the intervention ($1.50), which is still a relatively high cost as the countries with the lowest GDP per capita would pay only a fraction of that price under Gavi's current co-financing model, then the ICER for the RSV maternal vaccine and mAb would fall below the 50% GDP per capita threshold in 46% (maternal vaccine) and 100% (mAb) of these countries. Further, maternal vaccine ICERs across countries at base price are roughly equivalent to the mAb ICER evaluated

For peer review only - http://bmjopen.bmj.com/site/about/guidelines.xhtml

at 300% of the base price. Appendix Table 2 includes a comparison of ICERs as a share of country GDP for alternative intervention scenarios.

[Insert Figure 1 here: Incremental cost-effectiveness ratios as a percentage of national GDP per capita, maternal vaccine]

[Insert Figure 2 here: Incremental cost-effectiveness ratios as a percentage of national GDP per capita, monoclonal antibody]

Discussion

Both RSV interventions are projected to be impactful across all countries under baseline assumptions. A maternal vaccine is projected to avert 12.65 thousand deaths and mAbs roughly two times more (27.69 thousand deaths averted) annually among children younger than six months of age. We note that our baseline assumptions for the maternal vaccine draw from a Phase 3 trial in which the primary endpoints were not met. As a result, maternal vaccine assumptions may be conservative compared to mAb assumptions, leading to lower overall impact of RSV maternal vaccines. Under alternative scenarios that consider both interventions with similar characteristics, we observe no substantial variation in impact. Under a minimal (30% efficacy and four months protection) and maximal (90% efficacy and six months protection) intervention characteristics scenario, both interventions are projected to avert roughly 84,900 and 356,000 deaths among children younger than six months of age across 131 countries, suggesting that efficacy and duration of protection are primary parameters for determining impact, reinforced by a similar study [32].

Unknowns around intervention delivery strategy and potential coverage implications create uncertainties; this is especially true for a novel intervention like a maternal vaccine. To further understand the potential implications of unknown parameters on a maternal vaccine impact, we evaluated the marginal gains in impact by incrementally changing the parameter values to mimic those used in the mAb baseline scenario. When changing maternal vaccine coverage assumptions to the mAb coverage values, the maternal

For peer review only

For peer review only - http://bmjopen.bmj.com/site/about/guidelines.xhtml

vaccine would prevent 22,000 additional deaths. Similarly, when changing both duration of protection and efficacy for maternal vaccine at baseline to the mAb baseline equivalent, maternal vaccine would avert an additional 150,000 deaths. As seen in Figure 3, the duration of protection is the most important factor for increasing impact (109,000 additional deaths averted).

[Insert Figure 3 here: Impact of change in key input parameter values on deaths averted.]

The cost per DALY averted under the baseline scenario for a maternal vaccine is more than three times that for mAbs ($1,342 versus $431). This is mainly driven by the modest vaccine efficacy and assumed duration of protection for the maternal vaccine as compared to mAbs. Under the maximum and minimum scenarios with high and low vaccine efficacy and duration of protection assumptions, the difference in the estimated ICERs between the two interventions is muted (Figure 4).

[Insert Figure 4 here: Average incremental cost-effectiveness ratios by country groups.]

Though it didn't meet the primary endpoint, the recent Phase 3 maternal vaccine trial shows promising impact on all-cause LRTI mortality [15]. If both future RSV interventions reduce all-cause LRTI mortality, our adjunct scenario shows more pronounced impact by averting more than a million all-cause LRTIs during the 10-year period. ICER estimates under this scenario were $896 for the maternal vaccine (range $34 to $7,602) and $889 for the mAb (range $33 to $7,608) per DALY averted across all countries, with 116 countries (69 Gavi-eligible and 47 non-Gavi countries) demonstrating ICERs less than 50% of their respective GDP per capita. We refrained from directly comparing these estimates to other scenarios as they use data sources [21] not comparable with the primary disease burden data [2] used in other scenarios.

There are several additional limitations worth citing. There is a dearth of RSV disease burden data, especially regarding the age distribution of disease in young infants in LMICs. Although we used the best published estimates of RSV disease burden in children [2], the literature is expanding rapidly. For example, studies from Zambia [40] and Argentina [41] highlight that community mortality and deaths

For peer review only

For peer review only - http://bmjopen.bmj.com/site/about/guidelines.xhtml

324 from RSV could be as high as 10% and 11% of all-cause deaths occurring among infants up to six months
325 of age. This highlights a large and underappreciated burden of RSV and would mean our estimates of
326 impact and effectiveness are conservative. Although we attempted to quantify the potential benefits of
327 RSV interventions with additional scenario analysis, lack of consistent input data coupled with poorly
328 established age distribution limits the comparability of our results across these scenarios. Collecting more
329 granular data on disease burden is critical to inform future studies [32].

330 The products evaluated in this study are not yet available in the market so other key parameters are
331 unknown. We assumed the same price for both interventions, which may not hold, as historical evidence
332 suggests mAbs are likely to be more expensive to produce than a vaccine [5]. This could have
333 considerable impact on the ICERs and comparisons between products. Nonetheless, our analysis shows
334 that the mAb is more cost-effective than a maternal vaccine at baseline efficacy and duration of protection
335 values, until a mAb reaches approximately three times the baseline price assumption. Gavi evaluated both
336 interventions for inclusion in its 2018 Vaccine Investment Strategy in anticipation of the potential
337 benefits, and they are expected to be included in the Gavi portfolio, subject to licensure, prequalification,
338 and affordability. In that case, the eligible Gavi countries would benefit from a considerable subsidy for
339 access and affordability, especially the countries with the lowest GPDs per capita. Further, the <50% of
340 GDP per capita thresholds used in this paper are non-specific measures of cost-effectiveness, especially
341 when intervention prices to be paid by individual Gavi-supported countries are not yet known. Country-
342 specific thresholds are recommended [42] but often do not exist for most LMICs. In the absence of
343 country-specific thresholds, we used a conservative metric uniform across all countries to define cost-
344 effectiveness.

345 Lastly, RSV infection is seasonal in many countries. We did not consider seasonal delivery in this
346 analysis. Seasonal intervention could potentially be a more cost-effective yet feasible strategy [31],
347 especially when using mAbs to selectively immunize children before the start of the RSV season.
348 Delivering maternal vaccine seasonally to pregnant woman in LMICs may be more challenging due to the

For peer review only

For peer review only - http://bmjopen.bmj.com/site/about/guidelines.xhtml

lack of a defined maternal vaccine delivery strategy. Future research should explore the feasibility of alternative delivery strategies.

Conclusions

RSV interventions evaluated in this study are projected to be impactful and cost-effective across many LMICs. Under the assumptions used, mAbs are comparatively more impactful and cost-effective than RSV maternal vaccines. However, we reiterate the uncertainty around several critical parameters that inform this finding. The emerging evidence of RSV's role in all LRTI deaths among young infants suggests our analyses of RSV burden averted may prove conservative and enhance the attractiveness of RSV interventions as important tools for curbing LRTI mortality in infants. As disease burden shifts toward neonates and very young children, RSV maternal immunization and mAbs offer the opportunity to protect young infants from disease. As RSV interventions complete clinical development and the intervention characteristics and market prices becomes more definitive, future analysis will provide additional clarity on the anticipated health and economic impacts of these interventions.

Funding

This work was funded by a grant from the Bill & Melinda Gates Foundation [OPP1088264], Seattle, WA, USA. The findings and conclusions contained within are those of the authors and do not necessarily reflect positions or policies of the Bill & Melinda Gates Foundation.

Acknowledgments

We would like to thank Susan Nazarro for her critical feedback on this manuscript.

Author contributions

For peer review only - http://bmjopen.bmj.com/site/about/guidelines.xhtml

CP, DH and RB conceptualized the study. RB and CP developed the model. RB performed the analysis. RB and CP wrote the first draft of the paper. DH and KR reviewed and edited the manuscript.

Declaration of interest statement

All authors declare no conflict of interest.

Ethics approval

Not required.

Data sharing statement

All data relevant to the study are included in the article or uploaded as supplementary information.

References

1. Troeger C, Forouzanfar M, Rao PC, Khalil I, Brown A, Swartz S, et al. Estimates of the global, regional, and national morbidity, mortality, and aetiologies of lower respiratory tract infections in 195 countries: a systematic analysis for the Global Burden of Disease Study 2015. Lancet Infect Dis. 2017;17:1133-61. doi: 10.1016/S1473-3099(17)30396-1.

2. Shi T, McAllister DA, O'Brien KL, Simoes EAF, Madhi SA, Gessner BD, et al. Global, regional, and national disease burden estimates of acute lower respiratory infections due to respiratory syncytial virus in young children in 2015: a systematic review and modelling study. Lancet. 2017;390:946-58. doi: 10.1016/S0140-6736(17)30938-8.

3. Child Health and Mortality Prevention Surveillance (CHAMPS) [Internet]. c2020 [cited 2020 Apr 15]. Available from: https://champshealth.org/.

For peer review only - http://bmjopen.bmj.com/site/about/guidelines.xhtml

4. O'Brien KL, Baggett HC, Brooks WA, Feikin DR, Hammitt LL, Higdon MM, et al. Causes of severe pneumonia requiring hospital admission in children without HIV infection from Africa and Asia: the PERCH multi-country case-control study. Lancet. 2019;394:757-79. doi: 10.1016/S0140-6736(19)30721-4.

5. Utrecht University. News [Internet]. First consortium of local manufacturers to make affordable biosimilars available for low income countries. 9 Mar 2016. Available from: https://www.uu.nl/en/news/first-consortium-of-local-manufacturers-to-make-affordable-biosimilars-available-for-low-income.

6. Higgins D, Trujillo C, Keech C. Advances in RSV vaccine research and development – a global agenda. Vaccine. 2016;34:2870-5. doi: 10.1016/j.vaccine.2016.03.109.

7. PATH. RSV vaccine and mAb snapshot [Internet]. Seattle: PATH; March 2020 [cited 2019 Apr 23]. Available from: https://www.path.org/resources/rsv-vaccine-and-mab-snapshot/.

8. MedImmune LLC. A study to evaluate the safety and efficacy of MEDI8897 for the prevention of medically attended RSV LRTI in healthy preterm infants. (MEDI8897 Ph2b). 2017. Available from: https://www.clinicaltrials.gov/ct2/show/NCT02878330?term=NCT02878330&rank=1. [Cited 1 January 2017].

9. Gavi, the Vaccine Alliance. 06a - Annex C: Respiratory syncytial virus investment case. In: Vaccine Investment Strategy Programme and Policy Committee Meeting. 18-19 Oct 2018. Available from: https://www.gavi.org/sites/default/files/document/ppc-meeting-18-19-october-2018---vis-06a---annex-c--respiratory-syncytial-virus-investment-casepdf.pdf.

10. Ochalek J, Lomas J, Claxton K. Cost per DALY averted thresholds for low- and middle-income countries: evidence from cross country data. F1000Research. 2017;6:1–51. doi: 10.7490/f1000research.1113912.1.

11. United Nations Department of Economic and Social Affairs, Population Division [Internet]. World Population Prospects 2019. c2019 [cited 2019 Dec 15]. Available from: https://population.un.org/wpp/.

For peer review only - http://bmjopen.bmj.com/site/about/guidelines.xhtml

12. Lawn JE, Blencowe H, Waiswa P, Amouzou A, Mathers C, Hogan D, et al. Stillbirths: rates, risk factors, and acceleration towards 2030. Lancet. 2016;387:587-603. doi: 10.1016/S0140-6736(15)00837-5.

13. World Health Organization (WHO). Preferred product characteristics for respiratory syncytial virus (RSV) vaccines. Geneva: WHO; 2017. Available from: http://www.who.int/immunization/documents/research/who_ivb_17.11/en/.

14. ClinicalTrials.gov [Internet]. Bethesda (MD): National Library of Medicine (US). 2020 June 17 - . A trial to evaluate the efficacy and safety of RSVpreF in infants born to women vaccinated during pregnancy; 2021 Feb 3 [cited 2021 Mar 12]. Available from: https://clinicaltrials.gov/ct2/show/NCT04424316

15. Novavax, Inc. New data from Novavax Phase 3 Prepare™ trial of ResVax™ presented at 2019 IDSOG Annual Meeting [press release]. 12 Aug 2019. Available from: https://ir.novavax.com/news-releases/news-release-details/new-data-novavax-phase-3-preparetm-trial-resvaxtm-presented-0#.

16. World Health Organization (WHO). Preferred product characteristics of monoclonal antibodies for passive immunization against respiratory syncytial virus (RSV). Geneva: WHO; 2020. Available from: https://www.who.int/immunization/research/ppc-tpp/PPC_RSV-MAbs_Draft_V-0.1-for-consultation.pdf?ua=1.

17. U.S. Agency for International Development (USAID) [Internet]. Demographic and Health Surveys. c2020 [cited 2020 Jun 8]. Available from: https://www.dhsprogram.com/.

18. UNICEF [Internet]. Antenatal care. c2019 [cited 2020 Jun 8]. Available from: https://data.unicef.org/topic/maternal-health/antenatal-care/.

19. World Health Organization (WHO). WHO antenatal care randomized trial: manual for the implementation of the new model. Geneva: WHO; 2002. Available from: https://apps.who.int/iris/handle/10665/42513.

For peer review only

For peer review only - http://bmjopen.bmj.com/site/about/guidelines.xhtml

20. World Health Organization (WHO) [Internet]. Data, statistics and graphics. c2020 [cited 2020 Jun 8]. Available from: https://www.who.int/immunization/monitoring_surveillance/data/en/.

21. Institute for Health Metrics and Evaluations (IHME) [Internet]. Global Burden of Disease Study 2017 (GBD 2017) Cause-Specific Mortality 1980-2017. c2019 [cited 2020 Jun 8]. Available from: http://ghdx.healthdata.org/record/ihme-data/gbd-2017-cause-specific-mortality-1980-2017.

22. Rudan I, O'Brien KL, Nair H, Liu L, Theodoratou E, Qazi S, et al. Epidemiology and etiology of childhood pneumonia in 2010: estimates of incidence, severe morbidity, mortality, underlying risk factors and causative pathogens for 192 countries. J Glob Health. 2013;3:10401. doi: 10.7189/jogh.03.010401.

23. ImmunizationEconomics.org [Internet]. Immunization Costing Action Network (ICAN). c2020 [cited 2020 Jun 8]. Available from: http://immunizationeconomics.org/ican-home.

24. Zhang S, Sammon PM, King I, Andrade AL, Toscano CM, Araujo SN, et al. Cost of management of severe pneumonia in young children: systematic analysis. J Glob Health. 2016;6:10408. https://pubmed.ncbi.nlm.nih.gov/27231544.

25. Institute for Health Metrics and Evaluations (IHME) [Internet]. Global Burden of Disease Study 2010 (GBD 2010) Disability Weights. c2019 [cited 2020 Jun 8]. Available from: http://ghdx.healthdata.org/record/ihme-data/gbd-2010-disability-weights.

26. Graham BS, Anderson LJ. Challenges and opportunities for respiratory syncytial virus vaccines. Curr Top Microbiol Immunol. 2013;372:391-404. doi: 10.1007/978-3-642-38919-1_20.

27. World Health Organization (WHO) [Internet]. Children Aged <5 Years with Pneumonia Symptoms Taken to a Healthcare Provider (%) (Child Health). c2017 [cited 2020 Jun 8]. Available from: https://apps.who.int/gho/data/node.imr.WHS4_106?lang=en.

28. Baral R, Fleming J, Khan S, Higgins D, Hendrix N, Pecenka C. Inferring antenatal care visit timing in low- and middle-income countries: methods to inform potential maternal vaccine coverage. PLoS ONE. 2020;15(8): e0237718. https://doi.org/10.1371/journal.pone.0237718

For peer review only

For peer review only - http://bmjopen.bmj.com/site/about/guidelines.xhtml

29. Clark A, and Sanderson C. Timing of children's vaccinations in 45 low-income and middle-income countries: an analysis of survey data. Lancet. 2009. 373 (9674):1543–1549. 2008;23(8):579.

30. Debellut F, Clark A, Pecenka C, Tate J, Baral R, Sanderson C, Parashar U. Re-evaluating the potential impact and cost-effectiveness of rotavirus vaccination in 73 Gavi countries: a modeling study. Lancet Glob Health. 2019. 7: e1164–74.

31. Cromer D, van Hoek AJ, Newall AT, Pollard AJ, Jit M. Burden of paediatric respiratory syncytial virus disease and potential effect of different immunisation strategies: a modelling and cost-effectiveness analysis for England. Lancet Public Health. 2017;2:e367-74. doi: 10.1016/S2468-2667(17)30103-2.

32. Li X, Willem L, Antillon M, Bilcke J, Jit M, Beutels P. Health and economic burden of respiratory syncytial virus (RSV) disease and the cost-effectiveness of potential interventions against RSV among children under 5 years in 72 Gavi-eligible countries. BMC Med. 2020;18:82. doi: 10.1186/s12916-020-01537-6.

33. Gavi, the Vaccine Alliance [Internet]. Eligibility and transition policy. c2019 [cited 2019 May 20]. Available from: https://www.gavi.org/about/programme-policies/eligibility-and-transition/.

34. Wei C, Wang S, Yang Y, Al E, Li Ruili. Cost-effectiveness analysis of traditional Chinese medicine (TCM) and western medicine therapeutic schemes for 297 cases of child respiratory syncytial virus pneumonia. Chin J Pr Pediatr. 2008;23(8):579.

35. Xu H, Han X, Huang J. Multi-attribute evaluation of the clinical effect differences between Chinese medicine and western medicine in the treatment of pediatric RSV pneumonia. J Pediatr TCM. 2013;(1):23-27.

36. Zhang T, Zhu Q, Zhang X. Clinical characteristics and direct medical cost of respiratory syncytial virus infection in children hospitalized in Suzhou, China. Pediatr Infect Dis J. 2014;33(4):337-41. https://pubmed.ncbi.nlm.nih.gov/24088730/.

For peer review only

For peer review only - http://bmjopen.bmj.com/site/about/guidelines.xhtml

37. Chan PWK, Abdel-Latif MEA. Cost of hospitalization for respiratory syncytial virus chest infection and implications for passive immunization strategies in a developing nation. Acta Paediatr. 2003;92:481-5. doi: 10.1111/j.1651-2227.2003.tb00582.x.

38. Bhuiyan MU, Luby SP, Alamgir NI, Homaira N, Sturm-Ramirez K, Gurley ES, et al. Costs of hospitalization with respiratory syncytial virus illness among children aged <5 years and the financial impact on households in Bangladesh, 2010. J Glob Health. 2017;7:10412. doi: 10.7189/jogh.07.010412.

39. Khuri-Bulos N, Williams JV, Shehabi AA, Faouri S, Al Jundi E, Abushariah O, et al. Burden of respiratory syncytial virus in hospitalized infants and young children in Amman, Jordan. Scand J Infect Dis. 2010;42:368-74. doi: 10.3109/00365540903496544.

40. Williams AL et al. RSV-associated respiratory death among Zambian infants. Poster presentation at: RSV18. 2018.

41. Caballero MT, Bianchi AM, Nuño A, Ferretti AJP, Polack LM, Remondino I, et al. Mortality associated with acute respiratory infections among children at home. J Infect Dis. 2019;219:358-64. doi: 10.1093/infdis/jiy517.

42. Bertram MY, Lauer JA, De Joncheere K, Edejer T, Hutubessy R, Kieny MP, et al. Cost-effectiveness thresholds: pros and cons. Bull World Health Organ. 2016;94:925-30. doi: 10.2471/BLT.15.164418.

For peer review only - http://bmjopen.bmj.com/site/about/guidelines.xhtml

Figure captions

Figure 1. Incremental cost-effectiveness ratios as a percentage of national GDP per capita, maternal vaccine.

Figure 2. Incremental cost-effectiveness ratios as a percentage of national GDP per capita, monoclonal antibody.

Figure 3. Impact of change in key input parameter values on deaths averted.

Figure 4. Average incremental cost-effectiveness ratios by country groups.

For peer review only

For peer review only - http://bmjopen.bmj.com/site/about/guidelines.xhtml

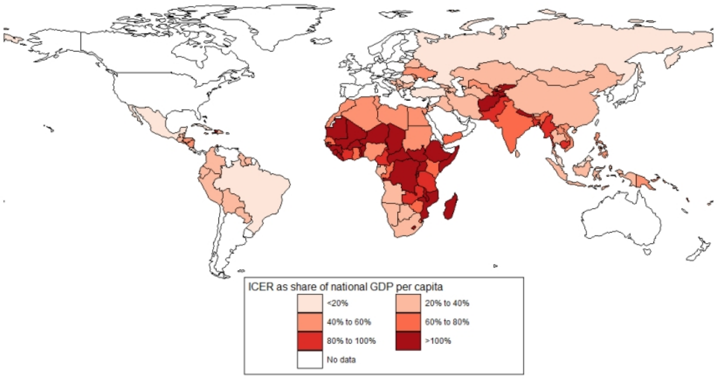

Figure 1: Incremental cost-effectiveness ratios as a percentage of national GDP per capita, maternal vaccine.

524x202mm (72 x 72 DPI)

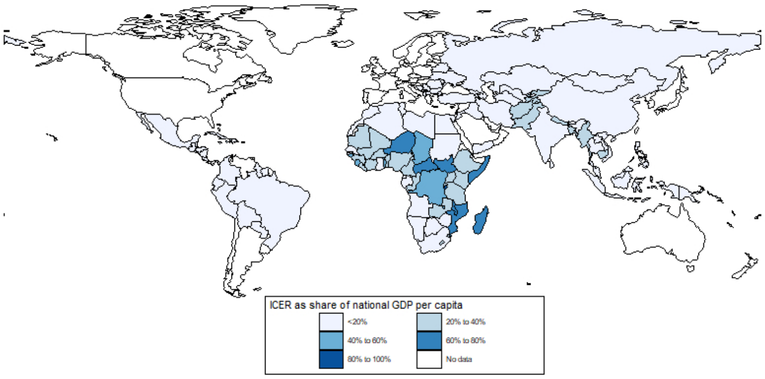

Figure 2: Incremental cost-effectiveness ratios as a percentage of national GDP per capita, monoclonal antibody.

358x138mm (72 x 72 DPI)

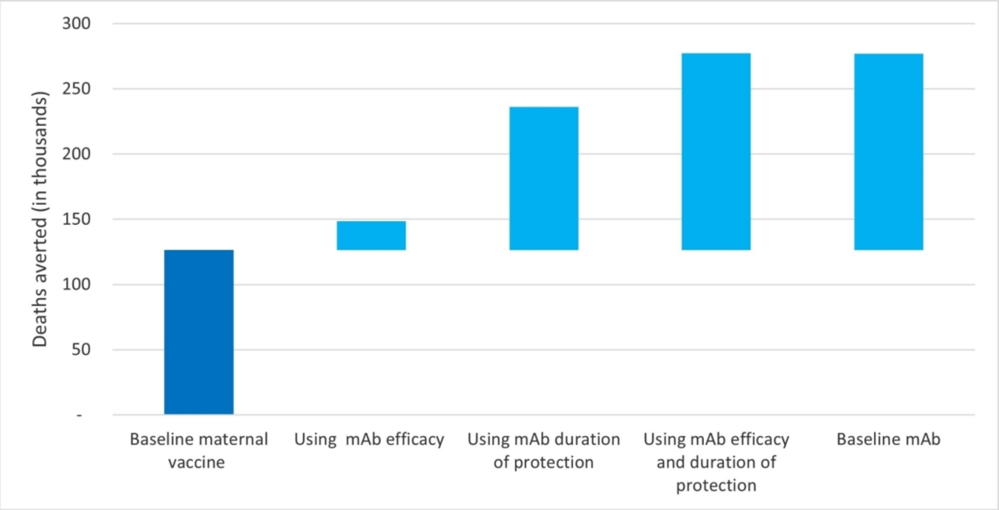

Figure 3. Impact of change in key input parameter values on deaths averted.

167x85mm (300 x 300 DPI)

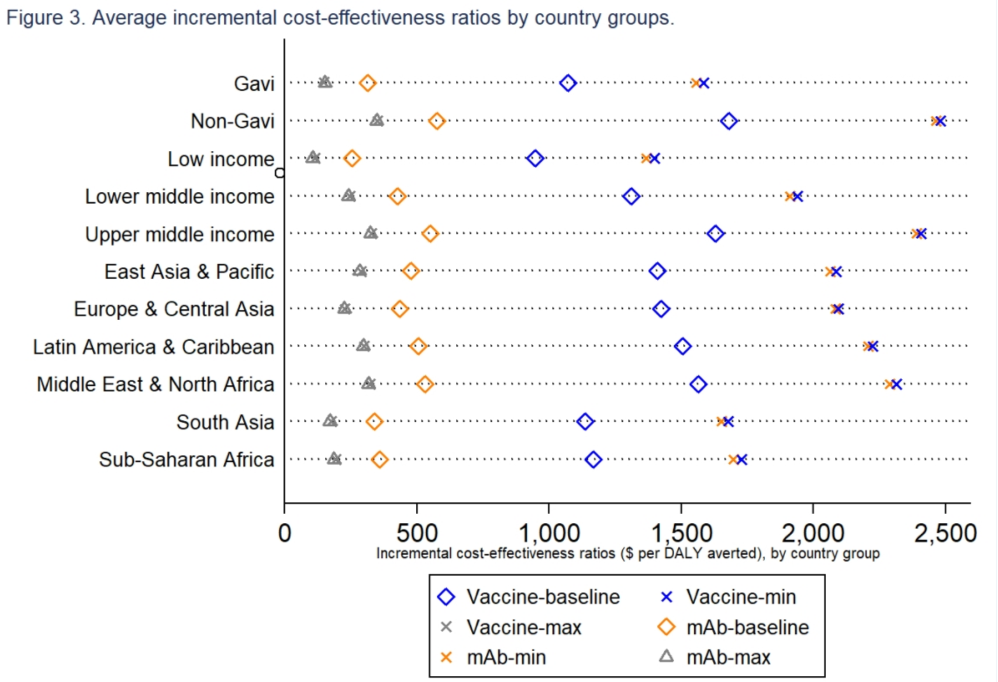

Figure 4 here: Average incremental cost-effectiveness ratios by country groups.

473x344mm (72 x 72 DPI)

Appendix Table 1. Cost of intervention and incremental cost-effectiveness ratios by country group (2030–2039).

Country group by	Maternal vaccine			Monoclonal antibody		
	Health care cost averted	Cost of vaccination program	ICER per DALY averted	Health care cost averted	Cost of vaccination program	ICER per DALY averted
Gavi status						
Gavi (N=73)	397,985,940	3,065,106,927	1,073	1,311,275,193	3,028,242,872	315
Non-Gavi (N=58)	204,118,914	2,398,496,379	1,681	663,227,127	2,355,785,836	577
World Bank Country income group						
LIC (N=34)	131,178,798	886,636,675	949	420,421,498	867,885,189	257
LMIC (N=46)	299,612,949	2,557,430,903	1,311	995,875,548	2,522,516,388	428
UMIC (N=51)	171,313,107	2,019,535,727	1,631	558,205,274	1,993,627,132	551
Geographic region						
East Asia & Pacific (N=20)	124,384,911	1,411,293,535	1,411	392,896,831	1,380,338,857	479
Europe & Central Asia (N=20)	32,220,016	301,556,780	1,425	109,878,013	295,554,675	437
Latin America & Caribbean (N=23)	46,562,615	515,567,727	1,507	146,732,622	492,924,565	507
Middle East & North Africa (N=13)	45,216,714	496,642,134	1,566	154,303,356	504,915,613	532
South Asia (N=8)	155,092,982	1,187,286,456	1,138	558,355,660	1,236,166,379	342
Sub-Saharan Africa (N=47)	198,627,617	1,551,256,674	1,169	612,335,839	1,474,128,619	359
Total (N=131)	602,104,854	5,463,603,306	1,342	1,974,502,320	5,384,028,709	431

For peer review only - http://bmjopen.bmj.com/site/about/guidelines.xhtml

Abbreviations: DALY, disability-adjusted life year; ICER, incremental cost-effectiveness ratio; LIC, low-income country; LMIC, low- and middle-income country; UMIC, upper-middle-income country.

Appendix Table 2. Incremental cost-effectiveness ratio per disability-adjusted life year (DALY) averted as a percentage of gross domestic product (GDP) per capita across various scenarios.

Scenarios		Maternal vaccine						Monoclonal antibody					
		Baseline	Baseline low price	Baseline high price	Minimum	Maximum	Adjunct	Baseline	Baseline low price	Baseline high price	Minimum	Maximum	Adjunct
Efficacy (%)		Trial (40%–60%)	Trial (40%–60%)	Trial (40%–60%)	30	90	Trial (25%–39%)	60%–70%	60%–70%	60%–70%	30	90	Trial (25%–39%)
Duration of Protection (months)		3	3	3	4	6	6	6	6	6	4	6	6
Intervention cost (Gavi/non-Gavi)		$3/$5	$1.5/$2.5	$6/$10	$3/$5	$3/$5	$3/$5	$3/$5	$1.5/$2.5	$6/$10	$3/$5	$3/$5	$3/$5
Afghanistan	1	163%	82%	325%	240%	16%	7%	41%	5%	113%	234%	15%	7%
Albania		37%	21%	69%	55%	6%	17%	11%	4%	26%	55%	6%	17%
Algeria		43%	25%	79%	64%	10%	35%	15%	7%	31%	63%	9%	35%
Angola	1	36%	23%	63%	54%	7%	4%	12%	6%	24%	53%	7%	4%
Armenia	1	31%	20%	55%	47%	6%	14%	10%	5%	21%	47%	6%	14%
Azerbaijan	1	30%	19%	52%	44%	5%	2%	9%	4%	19%	44%	5%	2%
Bangladesh	1	83%	52%	145%	123%	15%	8%	27%	13%	55%	122%	15%	8%
Belarus		32%	18%	59%	46%	4%	105%	9%	3%	21%	46%	4%	104%
Belize		34%	20%	63%	51%	7%	12%	12%	5%	25%	50%	7%	12%
Benin	1	122%	63%	240%	181%	20%	11%	37%	11%	90%	177%	19%	11%
Bhutan	1	40%	25%	70%	59%	7%	8%	12%	6%	26%	58%	7%	8%
Bolivia	1	36%	23%	63%	54%	6%	7%	11%	5%	23%	53%	6%	7%
Bosnia & Herzegovina		33%	19%	61%	48%	5%	156%	10%	3%	23%	48%	5%	156%
Botswana		26%	15%	47%	39%	7%	10%	10%	5%	19%	38%	7%	10%

Brazil		19%	11%	35%	28%	4%	11%	7%	3%	14%	28%	4%	11%
Bulgaria		22%	13%	40%	32%	4%	19%	7%	3%	15%	32%	4%	18%
Burkina Faso	1	158%	80%	315%	233%	17%	10%	43%	7%	114%	232%	17%	10%
Burundi	1	333%	168%	663%	490%	33%	27%	86%	12%	235%	484%	31%	27%
Cambodia	1	90%	56%	157%	133%	14%	7%	27%	12%	58%	132%	14%	7%
Cameroon	1	93%	59%	162%	139%	19%	11%	31%	16%	61%	134%	18%	11%
Cape Verde		56%	33%	102%	83%	13%	25%	20%	9%	40%	81%	12%	24%
C.A. Rep.	1	260%	133%	513%	386%	40%	9%	76%	20%	188%	373%	37%	9%
Chad	1	148%	76%	291%	220%	25%	7%	44%	13%	107%	211%	22%	7%
China		21%	12%	38%	31%	5%	13%	8%	4%	15%	31%	5%	13%
Colombia		28%	16%	51%	41%	6%	16%	10%	5%	21%	41%	6%	16%
Comoros	1	75%	38%	148%	111%	10%	9%	20%	4%	52%	105%	9%	9%
Congo	1	71%	45%	123%	106%	15%	14%	25%	13%	49%	106%	15%	14%
Costa Rica		14%	8%	26%	21%	3%	27%	5%	2%	10%	21%	3%	27%
Cuba	1	13%	8%	23%	18%	0%	22%	2%	0%	7%	19%	0%	22%
Côte d'Ivoire	1	86%	55%	150%	128%	16%	7%	28%	14%	57%	126%	16%	7%
N. Korea	1												
D. Rep. Congo	1	212%	109%	419%	314%	30%	14%	59%	14%	150%	301%	27%	13%
Djibouti	1	61%	38%	108%	90%	7%	11%	16%	5%	37%	88%	6%	11%
Dominican Republic		25%	14%	45%	37%	5%	8%	9%	4%	18%	36%	5%	8%
Ecuador		27%	16%	49%	40%	6%	10%	10%	5%	20%	40%	6%	10%
Egypt		50%	29%	92%	74%	11%	8%	18%	8%	37%	74%	11%	8%
El Salvador		44%	26%	81%	66%	9%	22%	15%	7%	32%	64%	9%	21%
Equatorial Guinea		20%	12%	37%	30%	5%	4%	7%	4%	15%	30%	5%	4%
Eritrea	1												
Ethiopia	1	127%	65%	251%	188%	18%	15%	37%	9%	93%	185%	17%	15%
Fiji		34%	20%	63%	51%	8%	9%	12%	6%	25%	50%	8%	9%
Gabon		26%	15%	47%	38%	5%	5%	9%	4%	18%	37%	5%	5%
Gambia	1	130%	65%	260%	191%	10%	19%	32%	3%	91%	190%	10%	18%
Georgia	1	30%	19%	53%	45%	5%	42%	9%	4%	20%	44%	5%	41%

Ghana	1	63%	40%	110%	94%	12%	11%	21%	10%	42%	92%	12%	11%
Grenada		17%	10%	31%	25%	4%	8%	6%	3%	12%	25%	4%	8%
Guatemala		40%	24%	74%	60%	9%	5%	15%	7%	30%	60%	9%	5%
Guinea	1	134%	68%	265%	199%	20%	8%	40%	10%	99%	197%	19%	8%
Guinea-Bissau	1	147%	75%	291%	218%	21%	20%	43%	10%	108%	215%	20%	19%
Guyana	1	25%	16%	44%	37%	3%	10%	7%	3%	15%	36%	3%	9%
Haiti	1	123%	62%	246%	181%	10%	8%	28%	1%	83%	174%	8%	8%
Honduras	1	47%	29%	82%	69%	7%	44%	14%	6%	30%	68%	7%	43%
India	1	65%	41%	114%	96%	9%	6%	19%	7%	41%	95%	8%	6%
Indonesia	1	32%	20%	57%	48%	5%	8%	10%	4%	21%	48%	5%	8%
Iran		32%	19%	59%	47%	7%	29%	11%	5%	23%	47%	7%	29%
Iraq		38%	22%	70%	56%	8%	18%	13%	6%	27%	55%	8%	18%
Jamaica		34%	20%	63%	51%	7%	36%	12%	5%	25%	50%	7%	35%
Jordan		41%	24%	74%	60%	8%	20%	13%	6%	29%	59%	8%	19%
Kazakhstan		22%	13%	41%	32%	4%	10%	7%	2%	15%	32%	3%	10%
Kenya	1	81%	51%	141%	120%	13%	8%	25%	11%	52%	118%	12%	8%
Kiribati	1	76%	48%	132%	113%	13%	23%	24%	12%	50%	112%	13%	23%
Kyrgyzstan	1	106%	67%	184%	157%	20%	34%	35%	17%	70%	156%	20%	33%
Lao PDR	1	51%	32%	88%	76%	10%	3%	16%	8%	33%	73%	9%	2%
Lebanon		19%	11%	34%	27%	4%	54%	6%	3%	13%	27%	4%	53%
Lesotho	1	121%	76%	211%	179%	19%	10%	36%	16%	76%	173%	18%	10%
Liberia	1	130%	66%	258%	192%	15%	17%	35%	7%	93%	189%	15%	16%
Libya		40%	24%	74%	60%	8%	109%	14%	6%	29%	59%	8%	108%
Madagascar	1	234%	120%	462%	346%	33%	14%	66%	16%	167%	335%	30%	14%
Malawi	1	307%	154%	614%	449%	21%	36%	70%	1%	207%	438%	18%	35%
Malaysia		17%	10%	32%	26%	4%	27%	6%	3%	13%	26%	4%	27%
Maldives		16%	9%	29%	23%	4%	52%	6%	3%	12%	23%	4%	52%
Mali	1	125%	65%	247%	186%	21%	12%	39%	11%	93%	183%	20%	12%
Mauritania	1	117%	74%	203%	174%	24%	15%	38%	19%	76%	167%	22%	15%
Mauritius		17%	10%	31%	25%	4%	18%	6%	3%	13%	25%	4%	18%
Mexico		20%	11%	36%	29%	4%	11%	7%	3%	14%	29%	4%	11%
Micronesia		55%	32%	100%	81%	13%	23%	20%	10%	40%	81%	13%	23%
Mongolia	1	33%	21%	57%	48%	6%	4%	11%	5%	21%	48%	6%	4%

Montenegro		23%	13%	42%	33%	3%	108%	7%	2%	15%	33%	3%	108%
Morocco		58%	34%	106%	85%	12%	24%	20%	9%	41%	84%	12%	24%
Mozambique	1	245%	124%	487%	361%	28%	34%	67%	12%	177%	360%	27%	34%
Myanmar	1	98%	62%	169%	144%	18%	9%	32%	16%	64%	144%	18%	9%
Namibia		39%	23%	72%	58%	9%	7%	14%	6%	29%	58%	9%	7%
Nepal	1	109%	54%	221%	160%	3%	11%	23%	-2%	73%	159%	3%	11%
Nicaragua	1	52%	33%	91%	77%	8%	15%	16%	7%	33%	76%	8%	15%
Niger	1	266%	135%	529%	392%	29%	20%	68%	10%	185%	379%	26%	19%
Nigeria	1	63%	40%	109%	93%	14%	3%	21%	11%	42%	91%	13%	3%
Pakistan	1	86%	54%	151%	127%	13%	9%	24%	10%	52%	120%	11%	8%
Papua New Guinea	1	51%	33%	89%	76%	10%	8%	17%	8%	34%	75%	10%	8%
Paraguay		31%	18%	57%	46%	6%	23%	10%	5%	22%	45%	6%	23%
Peru		27%	16%	50%	40%	6%	9%	10%	5%	20%	40%	6%	9%
Philippines		58%	34%	106%	85%	12%	10%	20%	9%	42%	85%	12%	10%
Republic of Moldova	1	58%	37%	102%	86%	9%	12%	18%	8%	38%	86%	9%	12%
Romania		17%	10%	31%	25%	3%	7%	5%	2%	12%	25%	3%	7%
Russian Federation		19%	11%	35%	28%	3%	25%	6%	2%	13%	28%	3%	25%
Rwanda	1	127%	65%	252%	188%	15%	16%	34%	6%	90%	183%	14%	15%
Saint Lucia		18%	10%	33%	26%	4%	13%	6%	3%	13%	26%	4%	13%
St. Vincent & Grenad.		23%	14%	43%	35%	5%	11%	8%	4%	17%	35%	5%	11%
Samoa		45%	26%	81%	66%	11%	26%	16%	8%	33%	65%	11%	26%
S. Tome & Principe	1	68%	43%	118%	100%	11%	13%	20%	9%	43%	98%	10%	13%
Senegal	1	73%	37%	146%	108%	7%	10%	19%	3%	52%	106%	7%	10%
Serbia		29%	17%	55%	43%	4%	114%	8%	3%	20%	43%	4%	113%
Sierra Leone	1	194%	97%	388%	285%	15%	11%	47%	3%	134%	282%	14%	10%
Solomon Islands	1	60%	38%	105%	89%	11%	15%	19%	9%	38%	86%	10%	15%
Somalia	1	217%	113%	425%	323%	41%	17%	69%	23%	161%	310%	38%	16%

South Africa		33%	19%	61%	49%	7%	8%	11%	5%	24%	48%	7%	7%
South Sudan	1	396%	196%	797%	580%	20%	14%	85%	-4%	264%	564%	17%	14%
Sri Lanka	1	30%	19%	52%	44%	6%	67%	10%	5%	20%	44%	6%	66%
State of Palestine		59%	35%	108%	89%	16%	124%	23%	12%	45%	88%	16%	123%
Sudan	1	51%	32%	89%	75%	9%	9%	16%	8%	33%	74%	9%	9%
Suriname		30%	18%	55%	44%	7%	10%	11%	5%	22%	44%	6%	10%
Swaziland		64%	38%	117%	95%	14%	7%	23%	11%	47%	95%	14%	7%
Syria													
Macedonia		31%	18%	57%	45%	4%	45%	9%	3%	21%	45%	4%	44%
Tajikistan	1	116%	60%	230%	172%	16%	7%	34%	8%	85%	171%	16%	7%
Thailand		28%	16%	51%	41%	6%	32%	10%	5%	20%	41%	6%	32%
Timor-Leste	1	60%	38%	104%	89%	11%	7%	19%	9%	38%	86%	10%	7%
Togo	1	162%	82%	321%	239%	19%	22%	44%	8%	115%	234%	18%	21%
Tonga		47%	28%	86%	70%	11%	24%	17%	8%	35%	69%	11%	23%
Tunisia		46%	27%	84%	68%	10%	83%	16%	8%	33%	67%	10%	83%
Turkey		15%	9%	27%	22%	3%	25%	5%	2%	11%	22%	3%	25%
Turkmenistan		27%	16%	50%	40%	5%	4%	9%	4%	19%	40%	5%	4%
Uganda	1	157%	78%	315%	230%	9%	29%	35%	0%	105%	224%	8%	29%
Ukraine	1	52%	33%	90%	76%	8%	89%	16%	7%	33%	76%	8%	89%
Tanzania	1	103%	52%	203%	151%	12%	10%	27%	5%	72%	146%	11%	10%
Uzbekistan	1	56%	35%	97%	83%	10%	5%	18%	9%	37%	82%	10%	5%
Vanuatu		62%	37%	113%	92%	15%	10%	23%	11%	46%	91%	15%	10%
Venezuela													
Viet Nam	1	51%	32%	89%	75%	9%	21%	16%	8%	34%	75%	9%	21%
Yemen	1	69%	35%	137%	102%	10%	13%	20%	5%	50%	100%	9%	13%
Zambia	1	99%	62%	173%	146%	15%	11%	29%	13%	62%	143%	14%	11%
Zimbabwe	1	74%	37%	146%	109%	9%	5%	21%	4%	54%	108%	9%	5%

Note: Country by Gavi status (Gavi country = 1). Current GDP values were not available for 4 countries (Venezuela, Syria, Eretria, North Korea) and were excluded from the analysis.

*Negative cost-effectiveness ratio implies cost savings.

Cells in green indicate lower ICER to GDP ratio and cells in red indicate higher ICER to GDP ratio.

For peer review only

CHEERS Checklist
Items to include when reporting economic evaluations of health interventions

The **ISPOR CHEERS Task Force Report**, *Consolidated Health Economic Evaluation Reporting Standards (CHEERS)—Explanation and Elaboration: A Report of the ISPOR Health Economic Evaluations Publication Guidelines Good Reporting Practices Task Force*, provides examples and further discussion of the 24-item CHEERS Checklist and the CHEERS Statement. It may be accessed via the *Value in Health* or via the ISPOR Health Economic Evaluation Publication Guidelines – CHEERS: Good Reporting Practices webpage: http://www.ispor.org/TaskForces/EconomicPubGuidelines.asp

Section/item	Item No	Recommendation	Reported on page No/ line No
**Title and abstract**			
Title	1	Identify the study as an economic evaluation or use more specific terms such as "cost-effectiveness analysis", and describe the interventions compared.	1
Abstract	2	Provide a structured summary of objectives, perspective, setting, methods (including study design and inputs), results (including base case and uncertainty analyses), and conclusions.	2
**Introduction**			
Background and objectives	3	Provide an explicit statement of the broader context for the study. Present the study question and its relevance for health policy or practice decisions.	4
**Methods**			
Target population and subgroups	4	Describe characteristics of the base case population and subgroups analysed, including why they were chosen.	5, 7
Setting and location	5	State relevant aspects of the system(s) in which the decision(s) need(s) to be made.	5
Study perspective	6	Describe the perspective of the study and relate this to the costs being evaluated.	5
Comparators	7	Describe the interventions or strategies being compared and state why they were chosen.	5
Time horizon	8	State the time horizon(s) over which costs and consequences are being evaluated and say why appropriate.	5
Discount rate	9	Report the choice of discount rate(s) used for costs and outcomes and say why appropriate.	10
Choice of health outcomes	10	Describe what outcomes were used as the measure(s) of benefit in the evaluation and their relevance for the type of analysis performed.	5
Measurement of effectiveness	11a	*Single study-based estimates:* Describe fully the design features of the single effectiveness study and why the single study was a sufficient source of clinical effectiveness data.	NA

For peer review only

	11b	*Synthesis-based estimates:* Describe fully the methods used for identification of included studies and synthesis of clinical effectiveness data.	9
Measurement and valuation of preference based outcomes	12	If applicable, describe the population and methods used to elicit preferences for outcomes.	NA
Estimating resources and costs	13a	*Single study-based economic evaluation:* Describe approaches used to estimate resource use associated with the alternative interventions. Describe primary or secondary research methods for valuing each resource item in terms of its unit cost. Describe any adjustments made to approximate to opportunity costs.	NA
	13b	*Model-based economic evaluation:* Describe approaches and data sources used to estimate resource use associated with model health states. Describe primary or secondary research methods for valuing each resource item in terms of its unit cost. Describe any adjustments made to approximate to opportunity costs.	9,10
Currency, price date, and conversion	14	Report the dates of the estimated resource quantities and unit costs. Describe methods for adjusting estimated unit costs to the year of reported costs if necessary. Describe methods for converting costs into a common currency base and the exchange rate.	5
Choice of model	15	Describe and give reasons for the specific type of decision-analytical model used. Providing a figure to show model structure is strongly recommended.	5
Assumptions	16	Describe all structural or other assumptions underpinning the decision-analytical model.	7-9
Analytical methods	17	Describe all analytical methods supporting the evaluation. This could include methods for dealing with skewed, missing, or censored data; extrapolation methods; methods for pooling data; approaches to validate or make adjustments (such as half cycle corrections) to a model; and methods for handling population heterogeneity and uncertainty.	9-10
**Results**			
Study parameters	18	Report the values, ranges, references, and, if used, probability distributions for all parameters. Report reasons or sources for distributions used to represent uncertainty where appropriate. Providing a table to show the input values is strongly recommended.	12-14
Incremental costs and outcomes	19	For each intervention, report mean values for the main categories of estimated costs and outcomes of interest, as well as mean differences between the comparator groups. If applicable, report incremental cost-effectiveness ratios.	13-14
Characterising uncertainty	20a	*Single study-based economic evaluation:* Describe the effects of sampling uncertainty for the estimated incremental cost and incremental effectiveness parameters, together with the impact	NA

For peer review only

		of methodological assumptions (such as discount rate, study perspective).	
	20b	*Model-based economic evaluation:* Describe the effects on the results of uncertainty for all input parameters, and uncertainty related to the structure of the model and assumptions.	14-16
Characterising heterogeneity	21	If applicable, report differences in costs, outcomes, or cost-effectiveness that can be explained by variations between subgroups of patients with different baseline characteristics or other observed variability in effects that are not reducible by more information.	Figures 3-4, Appendix table 2
**Discussion**			
Study findings, limitations, generalisability, and current knowledge	22	Summarise key study findings and describe how they support the conclusions reached. Discuss limitations and the generalisability of the findings and how the findings fit with current knowledge.	15-17
**Other**			
Source of funding	23	Describe how the study was funded and the role of the funder in the identification, design, conduct, and reporting of the analysis. Describe other non-monetary sources of support.	18
Conflicts of interest	24	Describe any potential for conflict of interest of study contributors in accordance with journal policy. In the absence of a journal policy, we recommend authors comply with International Committee of Medical Journal Editors recommendations.	19

For consistency, the CHEERS Statement checklist format is based on the format of the CONSORT statement checklist

The **ISPOR CHEERS Task Force Report** provides examples and further discussion of the 24-item CHEERS Checklist and the CHEERS Statement. It may be accessed via the *Value in Health* link or via the ISPOR Health Economic Evaluation Publication Guidelines – CHEERS: Good Reporting Practices webpage: http://www.ispor.org/TaskForces/EconomicPubGuidelines.asp

The citation for the CHEERS Task Force Report is:
Husereau D, Drummond M, Petrou S, et al. Consolidated health economic evaluation reporting standards (CHEERS)—Explanation and elaboration: A report of the ISPOR health economic evaluations publication guidelines good reporting practices task force. Value Health 2013;16:231-50.

For peer review only
